# Pattern of Diversity and Prediction of Suitable Areas of Grasshoppers from the Qinghai–Tibet Plateau in China (Orthoptera: Acridoidea)

**DOI:** 10.3390/insects16020191

**Published:** 2025-02-10

**Authors:** Bowen Bao, Xicheng Wang, Zhenrui Peng, Qingyao Zhu, Xinjiang Li, Daochuan Zhang

**Affiliations:** Key Laboratory of Zoological Systematics and Application of Hebei Province, College of Life Sciences, Hebei University, Baoding 071002, China; bbw2393926515@163.com (B.B.); 17355576307@163.com (X.W.); pengzhenrui1998@163.com (Z.P.); 18716176945@163.com (Q.Z.)

**Keywords:** grasshopper diversity, species diversity, Qinghai–Tibet Plateau, climate change, MaxEnt model

## Abstract

The Qinghai–Tibet Plateau is recognized as a biodiversity hotspot, with a wide variety of grasshopper species, including several endemic to the region, which play significant roles in both agricultural and forestry ecosystems. The purpose of this study was to analyze the species diversity and distribution pattern of grasshoppers on the Qinghai–Tibet Plateau. A comprehensive database comprising 390 grasshopper species was established through specimen collection, a literature review, and a geographical distribution data analysis. Diversity analysis showed that the diversity of species under the five vegetation types was relatively average. However, the alpine cold vegetation of Qinghai–Tibet and subtropical evergreen broad-leaved forest still showed a relatively high Shannon index and Simpson index. Grasshopper species are mainly concentrated in the eastern and southern parts of the Qinghai–Tibet Plateau. The richness pattern showed that grasshopper species diversity was particularly high in certain mountain areas, with Bayankala Mountain and Hengduan Mountain being endemic hotspots. The MaxEnt models were used to assess the potential habitats for four dominant genera of grasshoppers under projected climate change scenarios for 2050 and 2070. Altitude was the factor affecting the distribution of *Locusta*, *Chorthippus*, and *Kingdonella*, while precipitation and temperature were the factors affecting the distribution of *Leuconemacris*. These findings improve our understanding of the distribution patterns of different grasshopper species across various habitat types on the Qinghai–Tibet Plateau and provide valuable insights for developing targeted ecological protection strategies in response to environmental changes.

## Simple Summary

This study explores the diversity and distribution of different grasshopper species across the Qinghai–Tibet Plateau, a region renowned for its rich diversity. Through the compilation and analysis of grasshopper specimens, the literature, and geographic distribution data, we have created a species database that details the diversity and distribution of 390 species. The research highlights that the Tibetan Plateau’s endemic species are predominantly found in the Bayan Har Mountains and the Hengduan Mountains. We also predict changes in suitable habitats for grasshoppers under future climate scenarios, providing insights for pest management in agricultural landscapes and forests, as well as for biodiversity conservation.

## 1. Introduction

The Qinghai–Tibet Plateau, renowned as the ‘roof of the world’ and the ‘third pole’ is a highly biodiverse region with significant scientific research value. It also serves as an early warning zone and a sensitive area for global climate change. Issues such as glacier retreat, permafrost degradation, and a sharp decline in biodiversity have become increasingly prominent. These phenomena are warning us that human communities and natural ecosystems may face additional significant risks [1,2,3,4,5]. With a total area of 2,581,300 km^2^, the Qinghai–Tibet Plateau in China stands among the world’s biodiversity hotspots [6]. Due to the high altitude, diverse climatic conditions, and complex topography, the Qinghai–Tibet plateau provides a diverse range of habitats for insects. Many rare and primitive grasshopper species are exclusively distributed on the Qinghai–Tibet Plateau [7]. Grasshoppers refer to Orthoptera Acridoidea insects that exhibit rich species diversity worldwide—approximately 8270 species and 1244 subspecies [8]—occupying various niches within nature. They are more sensitive to changes in their habitats and play crucial roles as indicators for fluctuations in natural landscapes or habitats [9]. Currently, studies on grasshoppers within the Qinghai–Tibet Plateau primarily focus on compiling species lists and controlling a few harmful species, while investigations into their diversity and geographical distribution patterns remain scarce.

Grassland is the main ecological type on the Qinghai–Tibet Plateau, covering over 60% of its total area [10]. Grasshoppers are the typical phytophagous insects that harm grassland, and their diversity and distribution are closely related to the health and sustainable development of the ecosystem [11]. Relevant studies have shown that grasshopper outbreaks are closely related to climatic conditions [12]. Global warming increases the frequency of grasshopper outbreaks, leading to detrimental effects on agricultural production [13,14]. For example, warming oceans bring more cyclones that create more favorable breeding conditions for grasshoppers, leading to a 2020 plague on the East African continent not seen in decades [15]. Agricultural planting zones on the Qinghai–Tibet Plateau are mostly located in river valleys, which are the main zones for grasshopper breeding and activities [16]. The maximum entropy model (MaxEnt) was first introduced into the prediction of species distribution area by Steven et al. in 2004 [17]. With its strong prediction ability, MaxEnt is one of the commonly used prediction models for potential geographic distribution [18]. According to the Fifth Assessment Report of the United Nations Intergovernmental Panel on Climate Change, the global climate will continue to warm, and, compared with 1986–2005, by 2100, the global climate will increase by 0.3 °C to 4.5 °C [19]. Global warming may lead to changes in the habitat areas of species. In the current context, some researchers have used this model to investigate the potential spatial distribution pattern of grasshoppers in various regions [20,21,22], but no studies have been conducted to predict the suitable grasshopper areas in the Qinghai–Tibet Plateau under climate change scenarios based on the MaxEnt model.

The Tibetan Plateau is a biodiversity hotspot of global significance, home to a wealth of grasshopper species. In this study, the species diversity, species richness pattern, and endemic distribution pattern of grasshopper in the Qinghai–Tibet Plateau were analyzed by using the information on grasshopper species distribution recorded in specimens and the literature. Then, we extracted two widely distributed genera from the Qinghai–Tibet Plateau, i.e., *Locusta* and *Chorthippus*, and two endemic genera, i.e., *Leuconemacris* and *Kingdonella*, and predicted their current and future suitable habitats (2050, 2070). The aim of our study was as follows: (1) to reveal the species diversity and distribution pattern of grasshoppers on the Qinghai–Tibet Plateau; (2) to identify the endemic distribution area of grasshoppers on the Qinghai–Tibet Plateau; and (3) to predict the habitat of grasshoppers on the Qinghai–Tibet Plateau. In addition, to assess the potential impacts of future climate change on this habitat, we conducted an analysis of the biogeographical characteristics of grasshoppers and their interactions with the environment in this region. The analysis above provides a theoretical foundation and practical guidance for developing effective grasshopper management strategies and conserving biodiversity.

## 2. Materials and Methods

### 2.1. Determination of the Research Area

**Figure 1:**
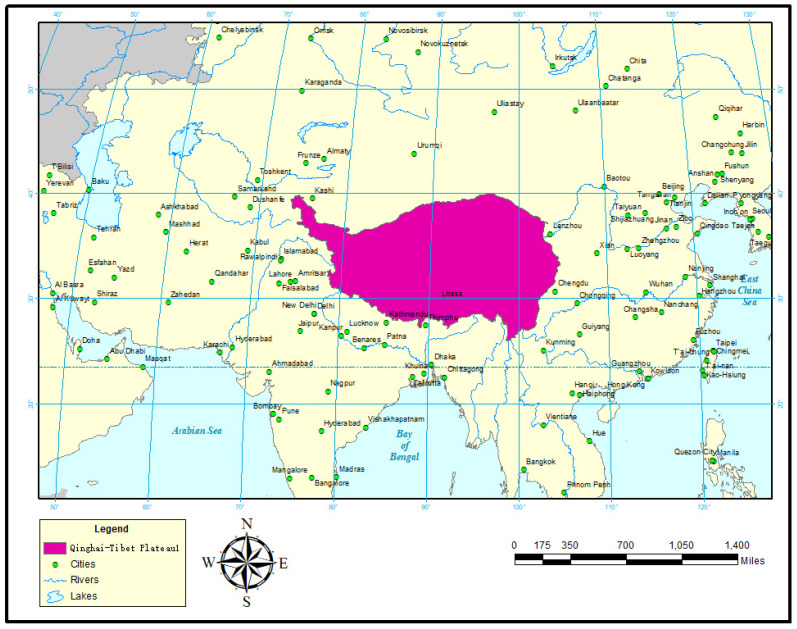
The Qinghai–Tibet Plateau and its external boundaries. This figure illustrates the geographic extent of the Qinghai–Tibet Plateau, marked in red, showcasing its position within the regional topography. The delineated area represents the plateau, and the boundaries distinguish it from adjacent landscapes. The map provides a visual overview of the plateau’s location within its broader geographical context, highlighting its significance as a distinct geographical feature.

Located in South–Central Asia, the Qinghai–Tibet Plateau is the largest plateau in China and the highest plateau in the world. It covers six provinces in southwest China: Tibet, Qinghai, Xinjiang, Gansu, Sichuan, and Yunnan. Lóczy [23] described the boundary of the Qinghai–Tibet Plateau, but the extent and specific boundary of the Tibetan Plateau have been the focus of controversy among many scholars [24]. The research area of this study was based on the 2021 Tibetan Plateau Range dataset, the results of the second comprehensive Scientific expedition to the Tibetan Plateau [25], which was downloaded from the Data Center for Resources and Environmental Sciences, Chinese Academy of Sciences (http://www.resdc.cn, accessed on 20 August 2024). The clipping function of ArcGIS 10.8 was used to convert the dataset into the Qinghai–Tibet Plateau region within China (Figure 1).

### 2.2. Data Collection and Organization

The inspected specimen data collected in the field were used for species diversity analysis under different habitat types. The data of species geographic distribution were used to analyze the distribution pattern of abundance and endemic species and to predict the suitable areas (Table 1, Appendix F). The geographical distribution data were partly obtained from global biodiversity information websites (https://www.gbif.org/, accessed on 20 August 2024) and Orthoptera Species File (Version 5.0/5.0) records (http://orthoptera.archive.speciesfile.org/Common/editTaxon/SearchForTaxon.aspx, accessed on 20 August 2024), and partly from specimens stored in scientific research institutions in China. The samples are collected in the field from the Laboratory of Systematics and Molecular Adaptation Evolution of Hebei University, the Museum of Hebei University, and the Northwest Plateau Ecology Research Institute. The species information and geographical distribution information were verified and corrected, and the distribution information containing only the name of the collection site was also included in the geographical distribution database of Acridoidea on the Qinghai–Tibet Plateau after calibration. Data on environmental variables for projections of suitable areas are available at 30 arc seconds (1 km^2^) from the World Climate website (http://www.worldclim.org, accessed on 20 August 2024). The vegetation normalization index was downloaded from the National Data Center for Ecological Sciences (http://www.nesdc.org.cn/, accessed on 20 August 2024) [26], with a resolution of 30 m. Potential evaporation and actual evaporation are downloaded at 30 arc seconds (1 km^2^) from the CGIAR website (http://www.cgiar.org/, accessed on 20 August 2024).

### 2.3. Species Diversity Analysis

The iNEXT program package in R 4.2.3 [27] was used to construct rarefaction curves under different habitat types, aiming to assess the adequacy of specimen collection. Vegetation type data were obtained from the Data Center for Resources and Environmental Sciences, Chinese Academy of Sciences (http://www.resdc.cn, accessed on 20 August 2024). According to the Vegetation Regionalization System of China, the Qinghai–Tibet Plateau was categorized into six habitat types: warm temperate evergreen broad-leaved forest, subtropical evergreen broad-leaved forest, tropical monsoon rainforest area, temperate grassland area, temperate desert area, and alpine vegetation area of Qinghai–Tibet Plateau [28]. The following diversity indices were selected for analysis using Past 4.1.3 software: Shannon–Wiener Index (H), Pielou Evenness Index (J), Margalef Richness Index (R), Simpson Index (D), and Berger–Parker Dominance Index (W). The Shannon Index is commonly employed to measure species diversity within an ecosystem by considering both species richness and evenness. The Pielou Evenness Index is a variant of the Shannon Index that quantifies the uniformity degree of species in a community. The Margalef Richness Index solely considers species count as a measure of species richness in a community. Like the Shannon Index, which measures species diversity within ecosystems, Simpson’s Diversity Index places greater emphasis on the abundance of common species. Lastly, the Berger–Parker Dominance Index measures the relative importance of the most dominant species in a community [29].

### 2.4. Analysis of Species Richness and Endemic Distribution Pattern

The entire Qinghai–Tibet Plateau region was divided into 0.5° × 0.5° grid geographic operational units. Following the principle of retaining only one piece of geographic distribution information for each species within a unit, the geographic distribution data in the database were manually sorted and screened. By using the ArcGIS 10.8 geographic operating system, we acquired patterns of species richness and endemic distribution for grasshoppers to determine their endemic distribution center on the Qinghai–Tibet Plateau. Two methods were used to identify endemic distribution areas at a 1° × 1° grid resolution: the unweighted average method and the endemic reductive analysis method. When using the unweighted mean group method to identify these areas based on data from endemic geographical records, a data matrix was established with geographic operational units as columns and species as rows. A value of “1” indicated presence while “0” indicated absence of a particular species in each unit. This matrix was then imported into Past 4.1.3 for cluster analysis [30] using the Jaccard similarity coefficient [31,32], with Bootstrap resampled 1000 times for testing purposes. According to Morrone’s definition [33] of an endemic distribution area, regions containing two or more identical endemic species in consecutive branches were considered. Only those with Bootstrap values greater than 60 were identified as endemic distribution areas.

To prepare a matrix for endemic reduction analysis, we selected a geographical operating unit with no species distribution to serve as an outgroup and named it “A0”. The characteristic reduction analysis was carried out in TNT1.1 software. Use new technical algorithms that have significant advantages in handling complex and large amounts of data [34]. The number of maximum trees was set to 1000, initial addseqs was set to 3, and the rest of the parameters were set to default. The optimal tree was searched using sectorial search and tree fusing tools. All generated parsimony trees were combined into a strict consensus tree. Branches with a bootstrap value of 50 or greater and that are geographically contiguous were selected as candidate branches for endemic distribution areas [35]. When two or more identical endemic species are present within these branches, the corresponding units are identified as endemic distribution areas.

### 2.5. Prediction of Suitable Areas

The geographical distribution data of the most widely distributed and most harmful grasshopper Genera *Locusta* and *Chorthippus*, as well as the endemic grasshopper genera *Leuconemacris* and *Kingdonella*, were extracted from the previously established geographical distribution database of grasshoppers on the Qinghai–Tibet Plateau. To reduce the distribution superposition and sampling bias, the overlapping data were discarded, and the systematic sampling method was applied to random sampling of species distribution information [36]. The environmental variables were selected as modern bioclimate factors, wind speed, solar radiation, actual evaporation, potential evaporation, vegetation normalization index, and altitude. Bioclimatic factors in the future period reduce the uncertainty between models by taking the mean value of MIROC-ESM and CCSM4 models [37]. Referring to previous studies, this paper selected bioclimatic factors under the scenario with a representative concentration path of 8.5 [38].

The software ENMTools v.1.3 was used to perform a Pearson correlation test to assess collinearity among all environmental variables [39]. When the correlation between two or more variables exceeded 0.85 (|r| > 0.85) [40,41,42], environmental factors with low contribution rates were eliminated using the Jackknife method. Relevant environmental variables with a cumulative contribution rate of 90% were then considered as the final set of environmental variables [43].

The selected species distribution data and modern environmental variable data were imported into MaxEnt version 3.4.3 software. Seventy-five percent of the distribution data were used as the training set, and 25% were used as the test set to construct the MaxEnt model for predicting species distributions. To prevent overfitting of the model, the combination of linear, quadratic, and hinge function feature types is selected [44,45,46]. The same parameter Settings were used to predict grasshopper habitats in 2050 and 2070, respectively. The prediction results were reclassified by ArcGIS 10.8, and the suitable areas were divided into four categories according to the probability range of species existence: non-suitable areas (0–0.2), low suitable areas (0.2–0.4), medium suitable areas (0.4–0.6), and high suitable areas (0.6–1) [2].

## 3. Results

### 3.1. Analysis of Species Composition

In this study, we compiled a species list of Acridoidea in the Qinghai–Tibet Plateau by consulting specimen records, records from the literature, and database records. The list includes 390 species from 121 genera and 4 families (see Appendix F). A total of 10,255 grasshopper specimens belonging to 4 families, 71 genera, and 168 species were included for the analysis to reveal the diversity structure of grasshopper communities in the region (see Appendix A).

The findings indicated that Acrididae accounted for a significant proportion (91.54%) of grasshoppers, highlighting its dominant position within the grasshopper community on the Qinghai–Tibet Plateau (Table 2). In terms of genus hierarchy, *Chorthippus* (Acrididae) emerged as an extremely dominant genus while *Indopodisma* (Acrididae), *Kingdonella* (Acrididae), and *Aeropus* (Acrididae) were identified as common genera. No specific dominant species were observed. Instead, there was a substantial presence of both common and rare species. This suggests high species diversity. It also indicates a relatively even distribution within the grasshopper community on the Qinghai–Tibet Plateau. Common species such as *Indopodisma kingdoni* (Acrididae), *Kingdonella bicollina* (Acrididae), and *Locusta migratoria migratorioides* (Acrididae) played crucial ecological roles within this community on the Tibetan Plateau, whereas rare species like *Catantops pinguis* (Acrididae), *Promesosternus vittatus* (Acrididae), and *Assamacris curticerca* (Acrididae) added complexity to enhance overall diversity within grasshopper communities on this plateau (Table 2).

### 3.2. Analysis of Species Diversity in Different Habitat Types

**Figure 2:**
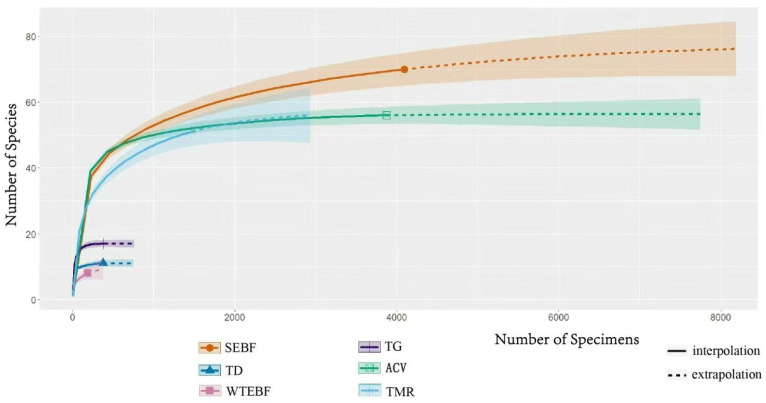
Sparse curves of Acridoidea in different habitats of the Qinghai–Tibet Plateau. This figure presents the species diversity indices of Acridoidea individuals randomly sampled from various habitats within the plateau using different algorithms in R studio. The y-axis represents the number of species, while the x-axis denotes the number of specimens. The point at which the dilution curves cease to show a significant upward trend indicates adequate sampling. Except for the evergreen broad-leaved forest in the warm temperate zone, which shows insufficient sampling, all other habitats demonstrate adequate sampling. Legend: SEBF—Subtropical Evergreen Broad-leaved Forest; TG—Temperate Grassland; TD—Temperate Desert; ACV—Alpine Cold Vegetation of Qinghai–Tibet; WTEBF—Warm Temperate Evergreen Broad-leaved Forest; TMR—Temperate Monsoon Rainforest.

The results of the species sampling adequacy test under different habitat types indicated that samples from all habitat types except for the warm temperate evergreen broad-leaved forest were deemed adequate (Figure 2). Therefore, the warm temperate evergreen broad-leaved forest habitat type was excluded, and the species diversity of grasshoppers in the remaining five habitat types was subsequently analyzed.

**Figure 3:**
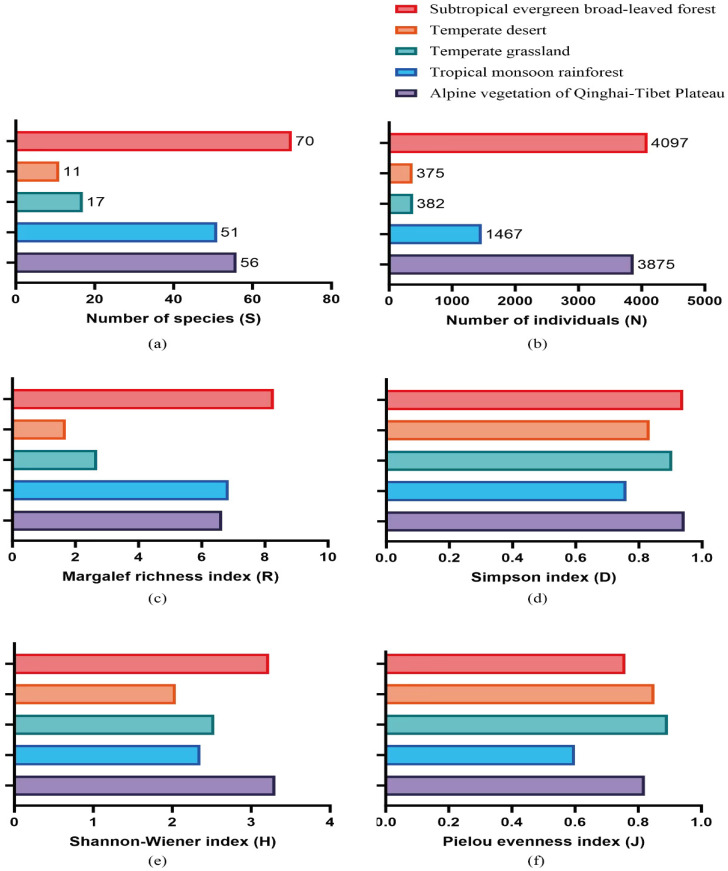
Alpha diversity index of Acridoidea in Qinghai–Tibet Plateau under different habitat types. Different habitat types; (**a**) number of species; (**b**) number of individuals; (**c**) Margalef Index (R); (**d**) Simpson Index (D); (**e**) Shannon–Wiener Index (H); (**f**) Pielou Index (J).

The diversity analysis results showed that the species and individual number were the most in the subtropical evergreen broad-leaved forest and the alpine vegetation area of the Qinghai–Tibet Plateau, while the species and individual number were the least in the temperate desert area (Figure 3a,b). The species richness index of subtropical evergreen broad-leaved forests was the highest (Figure 3c). The evenness index was the highest in temperate grassland areas, and the distribution of grasshopper species in this habitat was relatively well distributed, with no obvious dominant species (Figure 3f). The Shannon Index and the Simpson Index were the highest in the alpine vegetation area of the Qinghai–Tibet Plateau (Figure 3d,e). The Shannon Index considered species richness and evenness, and the Simpson Index considered species richness and relative abundance, indicating that the overall grasshopper diversity in this region was high. In summary, the alpine vegetation area and the subtropical evergreen broad-leaved forest on the Qinghai–Tibet Plateau show significantly high species diversity. In contrast, temperate desert areas have the lowest species diversity. The spatial distribution of grasshopper species was the most uniform in temperate grassland areas.

### 3.3. Distribution Pattern Analysis

**Figure 4:**
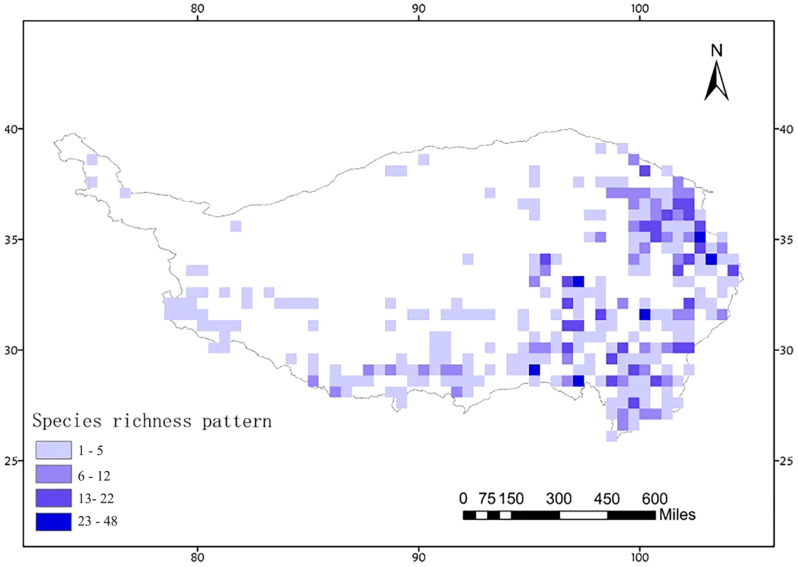
Species richness pattern of Acridoidea in the Qinghai–Tibet Plateau. This figure illustrates the distribution of species richness for Acridoidea across the Qinghai–Tibet Plateau, analyzed using ArcMap 10.8 software at a 0.5° grid scale. The species richness is categorized into four classes using the natural breaks (Jenks) classification method, with darker shades indicating a higher number of species within each grid. The data show a concentration of Acridoidea species in the eastern and southern regions of the plateau (between 94–104° E longitude and 26–34° N latitude), suggesting a pattern of higher species richness in these areas.

Based on the species distribution data of Acridoidea on the Qinghai–Tibet Plateau, a species distribution database of Acridoidea on the Qinghai–Tibet Plateau was constructed (see Appendix A for details). A total of 1540 distribution records of 390 species of 4 families were recorded, including 661 distribution records of 227 endemic species on the Qinghai–Tibet Plateau. We constructed the species richness pattern of Acidoidea on a 0.5° grid scale. The distribution of grasshopper species is mainly concentrated in the eastern and southern parts of the Qinghai–Tibet Plateau (94–104° E, 26–34° N), with the highest species richness in the eastern part of Qinghai Province, the Hengduan Mountains region, and the Metuo and Chaiyu regions of Tibet (Figure 4).

**Figure 5:**
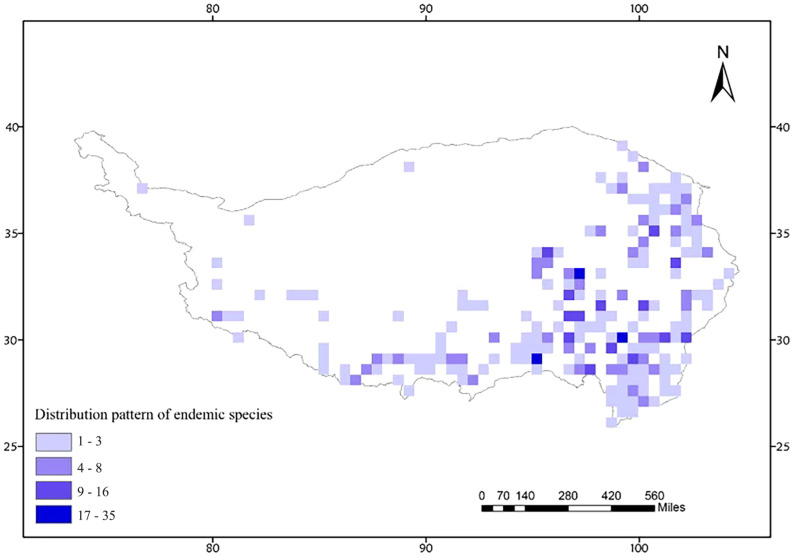
Species richness pattern of endemic species in the Qinghai–Tibet Plateau. This figure illustrates the distribution of endemic species richness across the Qinghai–Tibet Plateau, utilizing the same analytical methods as Figure 4.

**Figure 6:**
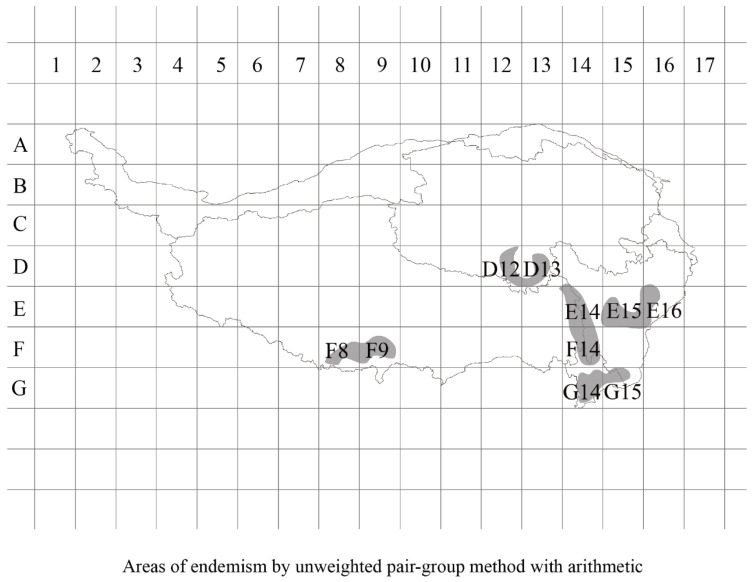

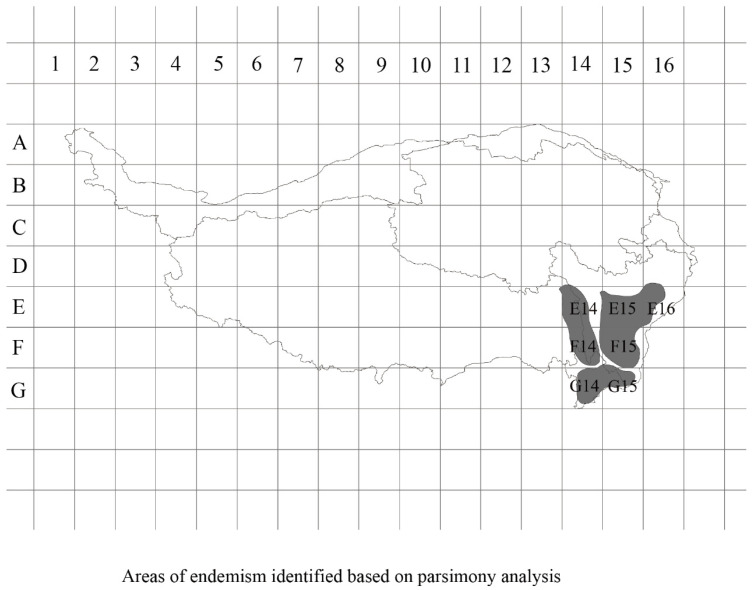
Specific distribution areas identified by two methods on a 1° grid scale. The non-weighted average group method reveals five endemic areas: Himalayas (F8, F9), Bayan Har Mountains (D12, D13), Jinsha River (E14, F14), Western Sichuan (E15, E16), and Hengduan Mountains (G14, G15). The endemicity parsimony analysis (PAE) identifies three areas: Jinsha River (E14, F14), Hengduan Mountains (G14, G15), and Western Sichuan Hengduan region (G14, G15). Both methods highlight the Jinsha River and Hengduan Mountains as key endemic regions, with the non-weighted method also noting the Himalayas and Bayan Har, and PAE marking Western Sichuan.

The distribution information of 227 endemic species and 661 geographic coordinates on the Tibetan Plateau was imported into ArcGIS 10.8 reoperating system to generate the distribution points of endemic species on the Tibetan Plateau, and the number of unique species in each 1° unit was counted to generate the distribution pattern of endemic species on the Tibetan Plateau (Figure 5). Based on the unweighted average method, five endemic areas were identified by consensus: the Himalayas (F8, F9), the Bayan Kala Mountains (D12, D13), the Jinsha River coast (E14, F14), the Western Sichuan (E15, E16), and the Hengduan Mountains (G14, G15) (Figure 6). Based on endemic reduction analysis, three endemic distribution areas were identified by consensus, namely, the Jinsha River coast (E14, F14), Hengduan Mountain Range (G14, G15), and Hengduan Mountain Range region in Western Sichuan (F15, E15, E16). The identified endemic species are shown in the attached table (see Appendix B and Appendix C).

### 3.4. Prediction of Suitable Areas

**Figure 7:**
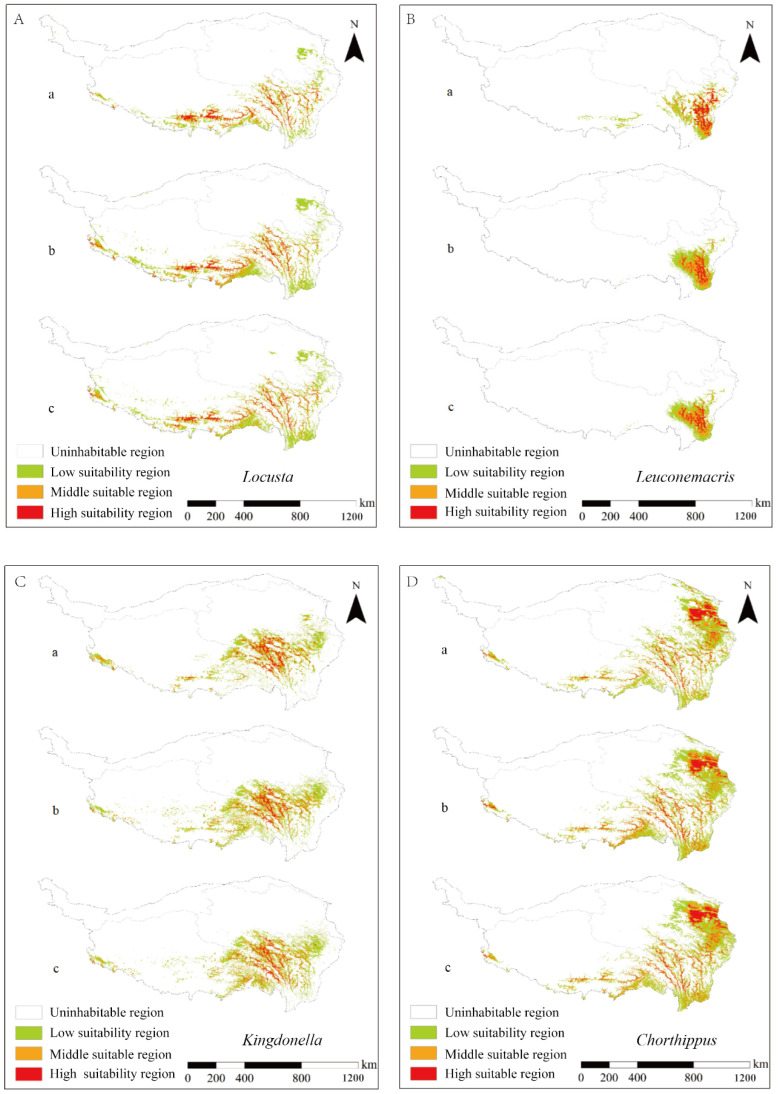
MaxEnt model predictions of suitable habitat areas for four genera of Acridoidea on the Qinghai–Tibet Plateau under current conditions (a), by 2050 (b), and by 2070 (c). The genera assessed are (**A**) *Locusta*, (**B**) *Leuconemacris*, (**C**) *Kingdonella*, and (**D**) *Chorthippus*. The model projections indicate shifts and changes in the distribution of suitable habitats for these genera over time, reflecting potential impacts of climate change on their biogeography.

In predicting the potential suitable habitats of four genera in the Qinghai–Tibet Plateau, relevant environmental factors were selected (see Appendix D). The MaxEnt model was employed to predict the potential distribution areas of four genera of Acrididae on the Qinghai–Tibet Plateau under three scenarios: current conditions (a), the RCP8.5 scenario for 2050 (b), and the RCP8.5 scenario for 2070 (c) (Figure 7). The AUC values exceeded 0.95, indicating a high degree of reliability in predictive performance. The results demonstrated varying degrees of influence from different environmental factors on the distribution patterns of each grasshopper genera. Notably, elevation was found to significantly impact the distribution of the *Locusta*. In contrast, annual precipitation, precipitation during the driest month, and seasonal temperature emerged as determinants affecting the distribution pattern of *Leuconemacris*. The distribution of *Kingdonella* was influenced by both elevation and wind speed, while that of *Chorthippus* exhibited a correlation with elevation and actual evaporation.

A comparative analysis between current and future suitable distribution areas revealed that all types within the *Locusta* displayed varying degrees of increase in suitable habitats alongside a trend toward expansion into lower elevations (see Appendix E). Although suitable regions for *Leuconemacris* within the Qinghai–Tibet Plateau are limited without an evident outbreak risk, Western Sichuan remains an area warranting close monitoring. The anticipated future suitable range for *Kingdonella* is expected to align closely with its current habitat preferences primarily located south of Bayanhar Mountains. Furthermore, evidence suggests a slight expansion in overall suitable areas for *Chorthippus*.

## 4. Discussion

This study provides a comprehensive analysis of the species composition of grasshoppers in the Qinghai–Tibet Plateau, revealing the diverse structure of grasshopper communities in this region. The findings reveal that, at the family level, Acrididae constitutes 95.84% of the individual grasshoppers. This finding aligns with previous observations regarding the dominant status of Acrididae in studies conducted on grasshopper fauna in Qinghai, China [47]. The large proportion of Acrididae in the Qinghai–Tibet Plateau can be attributed to the good adaptability of this family to high altitude, drought, and low temperature [48]. In contrast, Pamphagidae, Pyrgomorphidae, and Dericorythidae account for less than 5% of the species. In this study, *Chorthippus* was identified as an extremely dominant genus. *Chorthippus* species showed a good survival advantage in the study area, which was related to its excellent reproductive ability [49,50]. Genera such as *Indopodisma*, *Kingdonella*, and *Aeropus* are recognized as common genera. It must be pointed out here that we did not find dominant species in the Tibetan Plateau, which is different from what other scholars have found in investigating other ecosystem types [51,52,53]. Grasshoppers on the Qinghai–Tibet Plateau are mainly composed of many common and rare species. Rare species include species such as *Catantops pinguis*, *Assamacris curticerca*, and *Promesosternus vittatus*.

The formation of species diversity must be the result of the interaction of many environmental factors [54], and factors affecting species diversity vary in different regions [55]. This study reveals differences in the distribution of Acridoidea diversity in different habitats on the Qinghai–Tibet Plateau. The number of species and individuals in subtropical evergreen broad-leaved forests and alpine vegetation on the Qinghai–Tibet Plateau is the largest, which is related to the complexity of regional habitats and the richness of resources. The subtropical evergreen broad-leaved forest area provides diverse microhabitats and abundant food resources, creating favorable conditions for the survival and reproduction of Acridoidea [56,57]. Although the alpine vegetation area of the Qinghai–Tibet Plateau is restricted by temperature factors, it may promote the maintenance and enrichment of species diversity because it is less subjected to human disturbance [58,59]. Arid, barren, and highly fluctuating temperatures restrict the reproduction and survival of the Acridoidea species, which is the reason why temperate desert areas show the lowest species diversity, with the lowest number of species and individuals among all vegetation types [60].

The results of the distribution pattern indicate that the eastern and southern regions of the Qinghai–Tibet Plateau, particularly in eastern Qinghai Province, along the Hengduan Mountains, and in the Medog and Zayü areas of Tibet, are identified as hotspots for grasshopper species richness. This finding aligns with previous research on biodiversity within the Qinghai–Tibet Plateau [61]. Through unweighted average grouping methods and endemicity analysis, five endemic distribution areas have been identified that may represent significant geographical isolation zones crucial for species formation and divergence. Geographical isolation is recognized as a vital driver for speciation and evolution, especially under complex topographic features and varied climate conditions characteristic of the Qinghai–Tibet Plateau [62]. The endemic distribution areas located in the Himalayas, Bayanhar Mountain Range, Jinsha Riverbanks, Western Sichuan Province, and along the Hengduan Mountains facilitated both the formation and evolution of endemic species due to prolonged geographical isolation coupled with environmental heterogeneity [63,64].

For most environmental change scenarios, the negative impact on species richness in small hotspot areas is greater than the national average [64,65]. The Qinghai–Tibetan Plateau features a complex geological structure and exhibits sensitivity to climate change and human activities. Significant alterations in the fragile ecological balance of this region underscore the urgency of understanding and protecting its ecosystem quality [1]. Grasshoppers, as primary consumers within ecosystems, are widely utilized as biological indicators for studying the effects of global changes on these systems [66]. The MaxEnt model was used to predict potential distribution areas for four genera of grasshoppers in the Qinghai–Tibetan Plateau region for present conditions, as well as projections for 2050 and 2070. Results indicate that altitude and annual precipitation play critical roles in elucidating the relationship between grasshoppers and environmental factors. Similar findings were reported by Zhang (2024) in research conducted on grasshoppers in Ningxia Province, China [67]. Furthermore, previous large-scale studies have assessed multiple factors contributing to grasshopper population dynamics. These investigations revealed that grasshopper density is influenced by complex interactions among plant functional groups (PFG), nutrient litter (VT), and soil types (ST) [68]. Future research could enhance predictions regarding potential distribution areas of grasshoppers by incorporating additional environmental variables.

There are some limitations in this study, such as the temporal and spatial limitations in the sampling process, which failed to comprehensively observe the seasonal changes and spatial distribution patterns of grasshoppers. In the future, we need to further explore the dynamic relationship between grasshopper communities and environmental factors such as climate change, vegetation types, and human activities. Future research should combine long-term monitoring with multi-factor analysis to offer a more comprehensive assessment of grasshopper community conditions and species diversity on the Qinghai–Tibet Plateau.

## 5. Conclusions

The unique ecology of the Qinghai–Tibet Plateau promotes a diverse population of grasshoppers, and climate change may facilitate the expansion of their habitat. These findings underscore the imperative for targeted conservation initiatives in the identified priority areas, such as the Bayan Har Mountains and the Hengduan Mountains, to protect the region’s unique biodiversity and ecosystem services. The relative stability of the grasshopper community contrasts with the rapidly changing context of global biodiversity, highlighting the need to deepen our ecological understanding of the Qinghai–Tibet Plateau to predict and mitigate future changes to its unique ecosystems.

## Figures and Tables

**Table 1:** Distribution data of Acridoidea species in the Qinghai–Tibetan Plateau, China.

Family	Number of Distribution Records	Percentage of Total Family Distribution Records
Pamphagidae	7	0.45%
Pyrgomorphidae	32	2.08%
Dericorythidae	25	1.62%
Acrididae	1476	95.84%
Total	1540	100%

**Table 2:** Species composition of Acridoidea in the Qinghai–Tibetan Plateau.

Family	Number of Species	Percentage of Total Family	Number of Endemic Species
Pamphagidae	7	1.79%	1
Pyrgomorphidae	16	4.10%	10
Dericorythidae	10	2.56%	5
Acrididae	357	91.54%	205
Total	390	100%	227

## Data Availability

All the quantitative data analyzed are included in the Appendix A, Appendix B, Appendix C, Appendix D, Appendix E and Appendix F.

## Appendix A. Species List of Acridoidea in Qinghai-Tibetan Plateau

**Family**	**Genus**	**Species Name**
Pamphagidae	*Filchnerella*	*Filchnerella sunanensis* Liu, 1982
Pamphagidae		*Filchnerella zhengi* Huo, 1994
Pamphagidae		*Filchnerella rufitibia* Yin, 1984
Pamphagidae		*Filchnerella kukunoris* Bei-Bienko, 1984
Pamphagidae		*Filchnerella beicki* Ramme, 1931
Pamphagidae	*Eoeotmethis*	*Eoeotmethis longipennis* Zheng, 1985
Pamphagidae	*Beybienkia*	*Beybienkia amicus* (Bey-Bienko, 1959)
Pyrgomorphidae	*Aularches*	*Aularches miliaris* (Linnaeus, 1958)
Pyrgomorphidae	*Mekongiella*	*Mekongiella kingdoni* (Uvarov, 1937)
Pyrgomorphidae		*Mekongiella pleurodilata* Yin, 1984
Pyrgomorphidae		*Mekongiella rufitibia* Yin, 1984
Pyrgomorphidae		*Mekongiella xizangensis* Yin, 1984
Pyrgomorphidae		*Mekongiella wardi* (Uvarov, 1937)
Pyrgomorphidae	*Paramekongiella*	*Paramekongiella zhongdianensis* Huang, 1990
Pyrgomorphidae	*Mekongiana*	*Mekongiana gregoryi* (Uvarov, 1925)
Pyrgomorphidae		*Mekongiana xiangchengensis* Zheng, Huang&Zhou, 2008
Pyrgomorphidae	*Yunnanites*	*Yunnanites coriacea* Uvarov, 1925
Pyrgomorphidae		*Yunnanites zhengi* Mao&Yang, 2003
Pyrgomorphidae		*Yunnanites albomargina* Mao&Zheng, 1999
Pyrgomorphidae	*Tagasta*	*Tagasta indica* Bolívar, 1905
Pyrgomorphidae	*Atractomorpha*	*Atractomorpha melanostriga* Bi, 1981
Pyrgomorphidae		*Atractomorpha himalayica* Bolívar, 1905
Pyrgomorphidae		*Atractomorpha sinensis* Bolívar, 1905
Catantopidae	*Spathosternum*	*Spathosternum xizangensis* Yin, 1982
Acrididae	*Peripolus*	*Peripolus nepalensis* Uvarov, 1942
Acrididae	*Calliptamus*	*Calliptamus italicus* (Linnaeus,1758)
Acrididae		*Calliptamus barbarous* (Costa, 1836)
Acrididae		*Calliptamus abbreviates* Ikonnikov, 1913
Acrididae	*Xenocatantops*	*Xenocatantops brachycerus* (Willemse, 1932)
Acrididae		*Xenocatantops acanthraus* Zheng, Li&Wang, 2004
Acrididae		*Xenocatantops sauteri* (Ramme, 1941)
Acrididae	*Stenocatantops*	*Stenocatantops splendens* (Thunberg, 1815)
Acrididae		*Stenocatantops mistshenkoi* Willemse, 1968
Acrididae	*Catantops*	*Catantops simlae* Dirsh, 1956
Acrididae		*Catantops splendena* (Thunb)
Acrididae		*Catantops pinguis pinguis* (Stål, 1860)
Acrididae	*Schistocerca*	*Schistocerca gregaria* (Forskal, 1775)
Acrididae	*Patanga*	*Patanga japonica* (Bolívar, 1898)
Acrididae		*Patanga humilis* Bi, 1986
Acrididae	*Chondracris*	*Chondracris rosea brunneri* Uvarov, 1924
Acrididae	*Choroedocus*	*Choroedocus robusia* (Servile, 1839)
Acrididae	*Shirakiacris*	*Shirakiacris yunkweiensis* (Chang, 1937)
Acrididae		*Shirakiacris brachyptera* Zheng, 1983
Acrididae	*Eucoptacra*	*Eucoptacra moiuoensis* Yin, 1984
Acrididae	*Apalacris*	*Apalacris varicornis* Walker, 1870
Acrididae		*Apalacris xizangensis* Bi, 1984
Acrididae	*Ecphanthacris*	*Ecphanthacris mirabilis* Tinkham, 1940
Acrididae	*Assamacris*	*Assamacris longicercus* (Huang, 1981)
Acrididae		*Assamacris curticerca* (Huang, 1981)
Acrididae	*Promesosternus*	*Promesosternus himalayicus* Yin, 1984
Acrididae		*Promesosternus vittatus* Yin, 1984
Acrididae	*Habrocnemis*	*Habrocnemis sinensis* Uvarov, 1930
Acrididae	*Oxya*	*Oxya agavisa agavisa* Tsai, 1931
Acrididae		*Oxya chinensis* (Thunberg, 1815)
Acrididae		*Oxya intricata* (Stål, 1861)
Acrididae		*Oxya adentata* Willemse, 1925
Acrididae		*Oxya brachyptera* Zheng&Huo, 1992
Acrididae		*Oxya velox* (Fabricius, 1787)
Acrididae		*Oxya japonica* (Thunberg, 1824)
Acrididae	*Caryanda*	*Caryanda gyirongensis* Huang, 1981
Acrididae		*Caryanda bidentata* Zheng&Liang, 1985
Acrididae	*Parapodisma*	*Parapodisma astris* Huang, 2006
Dericorythidae	*Conophymacris*	*Conophymacris xianggelilaensis* Niu&Zheng, 2009
Dericorythidae		*Conophymacris yunnanensis* Willemse Cheng, 1977
Dericorythidae		*Conophymacris jiulongensis* Zheng&Shi, 2009
Dericorythidae		*Conophymacris cangshanensis* Zheng&Mao, 1996
Dericorythidae		*Conophymacris nigrofemora* Liang, 1993
Dericorythidae	*Sinopodisma*	*Sinopodisma zheng* Liang&Lin, 1995
Acrididae	*Curvipennis*	*Curvipennis wixiensis* (Yin,1980)
Acrididae	*Pedopodisma*	*Pedopodisma emeiensis* (Yin, 1980)
Acrididae		*Pedopodisma abaensis* Yin, Zheng&Ye, 2012
Acrididae	*Pseudozubovskia*	*Pseudozubovskia xizangensis* Zheng, Lin, Zhang&Zeng, 2014
Acrididae	*Xiangelilacris*	*Xiangelilacris zhongdianensis* Zheng, Huang&Zhou, 2008
Acrididae	*Rectimargipodisma*	*Rectimargipodisma medogensis* Zheng, Li&Wang, 2004
Acrididae	*Indopodisma*	*Indopodisma kingdoni* (Uvarov, 1927)
Acrididae	*Peseudoptygonotus*	*Peseudoptygonotus aipinus*
Acrididae	*Fruhstorferiola*	*Fruhstorferiola omei* (Rehn, 1939)
Acrididae	*Tylotropidius*	*Tylotropidius yunnanensis* Zheng er Liang, 1990
Acrididae	*Rhinopodisma*	*Rhinopodisma eminifrontus* (Huang, 1981)
Acrididae	*Eokingdonella*	*Eokingdonella changtunica* Yin, 1984
Acrididae		*Eokingdonella cyanecuda* Zheng&Shi, 2009
Acrididae		*Eokingdonella angqianensis* Chen&Zheng, 2009
Acrididae		*Eokingdonella gongbugyanda* Zheng, Lin, Zhang&Zeng, 2014
Acrididae		*Eokingdonella gentiana* (Uvarvo,1939)
Acrididae		*Eokingdonella tibetana* (Mistshenko, 1952)
Acrididae		*Eokingdonella burrusitibia* Zheng, Dong&Xu, 2013
Acrididae		*Eokingdonella kualbacki* (Uvarvo,1939)
Acrididae		*Eokingdonella bayanharaensis* (Huo, 1995)
Acrididae		*Eokingdonella luhuoensis* Zheng&Shi, 2007
Acrididae	*Kingdonella*	*Kingdonella qinghaiensis* Zheng, 1990
Acrididae		*Kingdonella nigrotibia* Zheng, 1990
Acrididae		*Kingdonella bicollina* Yin, 1984
Acrididae		*Kingdonella magna* Yin, 1984
Acrididae		*Kingdonella pienbaensis* Zheng, 1980
Acrididae		*Kingdonella nigrofemora* Yin, 1984
Acrididae		*Kingdonella hanburyi* Uvarov, 1939
Acrididae		*Kingdonella parvula* Yin, 1984
Acrididae		*Kingdonella longiconica* Yin, 1984
Acrididae		*Kingdonella pictipes* Uvarov, 1935
Acrididae		*Kingdonella kozlovi* Mistshenko, 1952
Acrididae		*Kingdonella conica* Yin, 1984
Acrididae		*Kingdonella afurcula* Yin, 1984
Acrididae		*Kingdonella sxicola* Uvarvo, 1939
Acrididae		*Kingdonellarivuna* Huang, 1981
Acrididae		*Kingdonella wardi* Uvarvo, 1939
Acrididae		*Kingdonella conica* Yin, 1984
Acrididae	*Anepipodisma*	*Anepipodisma punctata* Huang, 1984
Dericorythidae	*Conophymopsis*	*Conophymopsis linguspinus* Huang, 1983
Dericorythidae		*Conophymopsis labrispinus* Huang, 1983
Dericorythidae	*Diricorys*	*Diricorys annulata roseipennis* (Redtenbacher, 1889)
Acrididae	*Leptopternis*	*Leptopternis gracilis* (Eversmann, 1848)
Acrididae	*Heteropternis*	*Heteropternis respondens* (Walker, 1859)
Acrididae		*Heteropternis robusta* Bey-Bienko, 1951
Acrididae		*Heteropternis motuoensis* Yin, 1984
Acrididae		*Heteropternis qinghaiensis* Wang&Zheng, 1992
Acrididae		*Heteropternis micronus* Huang, 1981
Acrididae	*Longipternis*	*Longipternis chayuensis* (Yin, 1984)
Acrididae	*Mecostethus*	*Mecostethus grossus* (Linnaeus, 1758)
Acrididae	*Ochyracris*	*Ochyracris rufotibialis* Zheng, 1991
Acrididae	*Epacromius*	*Epacromius coerulipes* (Ivanov, 1888)
Acrididae		*Epacromius tergestinus tergestinus* (Megerle von Mühlfeld, 1825)
Acrididae		*Epacromius tergestinus extimus* Bey-Bienko, 1951
Acrididae	*Aiolopus*	*Aiolopus thalassinus* (Fabricius, 1798)
Acrididae		*Aiolopus tamulus* (Fabricius, 1798)
Acrididae		*Aiolopus markamensis* Yin, 1984
Acrididae	*Oedaleus*	*Oedaleus infernalis* Saussure, 1884
Acrididae		*Oedaleus asiaaicus* Bey-Bienko, 1941
Acrididae		*Oedaleus manjius* Chang, 1939
Acrididae	*Acrotylus*	*Acrotylus longipes subfasciatus* Bey-Bienko, 1984
Acrididae	*Aurilobulus*	*Aurilobulus splendens* Yin, 1979
Acrididae	*Cyanicaudata*	*Cyanicaudata annulicornea* Yin, 1979
Acrididae	*Celes*	*Celes skalozubovi* Adelung, 1906
Acrididae		*Celes akitanus* (Shiraki, 1910)
Acrididae	*Sphingonotu*	*Sphingonotus kueideensis* Yin, 1984
Acrididae		*Sphingonotus qinghaiensis* Yin, 1984
Acrididae		*Sphingonotus yunnaneus* Uvarov,1925
Acrididae		*Sphingonotus (Sphingonotus) tipicus* Zheng&Hang, 1974
Acrididae		*Sphingonotus halophilus* Bey-Bienko, 1929
Acrididae		*Sphingonotus tzaidamicus* Mistshenko,1937
Acrididae		*Sphingonotus caerulistriatus* Zheng&Ren,2007
Acrididae		*Sphingonotus zadaensis* Huang, 1981
Acrididae		*Sphingonotus longipennis* Saussure, 1884
Acrididae		*Sphingonotus turcmenus* Bey-Bienko, 1951
Acrididae	*Tibetacris*	*Tibetacris changtunensis* Chen, 1964
Acrididae		*Trilophidia annulata* (Thunberg, 1815)
Acrididae	*Parahilethera*	*Parahilethera xizangensis* Zheng&Ren, 2007
Acrididae	*Gastrimargus*	*Gastrimargus africanus africanus* (Saussure, 1888)
Acrididae		*Gastrimargus nubilis* Uvarov, 1925
Acrididae		*Gastrimargus marmoratus* (Thunberg, 1815)
Acrididae		*Gastrimargus ommatidius* Huang, 1981
Acrididae		*Gastrimargus africanus parvulus* Sjöstedt, 1928
Acrididae	*Locusta*	*Locusta migratoria tibetensis* Chen, 1963
Acrididae		*Locusta migratoria manilensis* (Meyen, 1835)
Acrididae		*Locusta migratoria migratoria* (Linnaeus, 1758)
Acrididae	*Pternoscirta*	*Pternoscirta saueri* (Karny, 1915)
Acrididae		*Pternoscirta pulchripes* Uvarov,1925
Acrididae		*Pternoscirta longipennis* Xia, 1981
Acrididae	*Bryodemella*	*Bryodemella xizangensis* Yin, 1984
Acrididae		*Bryodemella tuberculatum dilutum* (Stoll,1813)
Acrididae		*Bryodemella diamesum* (Bey-Bienko, 1930)
Acrididae		*Bryodemella (Bryodemella) rufifemura* Zhang, Zhi&Zhang, 2018
Acrididae		*Bryodemella (Bryodemella) nigrifemura* Yin&Wang, 2005
Acrididae		*Bryodemella holdereri holdereri* (Krauss, 1901)
Acrididae		*Bryodema luctuosum luctuosum (*Stoll, 1813)
Acrididae		*Bryodema miramae miramae* Bey-Bienko, 1930
Acrididae		*Bryodema uvarovi* (Bey-Bienko, 1930)
Acrididae		*Bryodema miramae elegantulum* Bey-Bienko, 1930
Acrididae		*Bryodema luctuosum inda* Saussure, 1884
Acrididae		*Bryodema hyalinala* Zheng&Zhang, 1981
Acrididae		*Bryodema pseudohyalinala* Zheng, Lin&Zhang, 2012
Acrididae		*Bryodema kangmarensis* Zheng, Lin&Zhang, 2012
Acrididae		*Bryodema nigristria* Zheng&Chen, 2001
Acrididae		*Bryodema gebleri gebleri* (Fischer von Waldheim, 1836)
Acrididae		*Bryodema gansuensis* Zheng, 1985
Acrididae		*Bryodema qilianshanicum* Lian&Zheng,1984
Acrididae	*Angaracris*	*Angaracris barabensis (Pallas, 1773)*
Acrididae		*Angaracris rhodopa* (Fischer von Waldheim, 1836)
Acrididae		*Angaracris morulimarginis* Huang, 1981
Acrididae		*Angaracris acrohylinna* Bi, 1986
Acrididae		*Angaracris nigripennis* Lian&Zheng, 1984
Acrididae	*Uvaroviola*	*Uvaroviola multispinosa* Bey-Bienko, 1930
Acrididae	*Ceracris*	*Ceracris nigricornis nigricornis* Walker, 1870
Acrididae		*Ceracris xizangensis xizangensis* Liu, 1981
Acrididae		*Ceracris versicolor* (Brunner von Wattenwyl, 1893)
Acrididae		*Ceracris fasciata* (Brunner-Wattenwyl, 1893)
Acrididae	*Ceracrisoides*	*Ceracrisoides shannanensis* Bi&Xia, 1987
Acrididae	*Anaptygus*	*Anaptygus qinghaiensis* Yin, 1984
Acrididae		*Anaptygus yueliangwan* Mao, Ren&Ou, 2011
Acrididae		*Anaptygus yulongensis* Wang, Zheng&Lian, 2005
Acrididae		*Anaptygus furculus* Huo&Zheng
Acrididae		*Anaptygus longipennis* Wang, Zheng&Lian, 2004
Acrididae		*Anaptygus uvarovi* (Chang, 1937)
Acrididae	*Arcyptera*	*Arcyptera coreana* Shiraki, 1930
Acrididae	*Leuconemacris*	*Leuconemacris litangensis* (Yin, 1983)
Acrididae		*Leuconemacris xizangensis* (Yin, 1984)
Acrididae		*Leuconemacris daochengensis* Zheng, 1988
Acrididae		*Leuconemacris longipennis* Zheng, 1988
Acrididae		*Leuconemacris breviptera* (Yin, 1983)
Acrididae		*Leuconemacris xiangchengensis* Zheng, 1988
Acrididae		*Leuconemacris shalulishanensis* Zheng, 1988
Acrididae		*Leuconemacris hengduanshanensis* Zheng, 1988
Acrididae		*Leuconemacris xinlongensis* Mao&Huang, 2023
Acrididae		*Leuconemacris acuminata* Mao&Huang, 2023
Acrididae		*Leuconemacris muliensis* Mao&Huang 2023
Acrididae	*Pararcyptera*	*Pararcyptera microptera* Ikonnikov, 1911
Acrididae	*Dnopherula*	*Dnopherula brevicornis* (Bi&Xia, 1987)
Acrididae		*Dnopherula taeniatus* (Bolívar, 1902)
Acrididae		*Dnopherula xizangensis* (Zheng&Liping, 1986)
Acrididae		*Dnopherula yuanmowensis* (Zheng, 1977)
Acrididae		*Dnopherula neovittatus* Otte, 1997
Acrididae		*Dnopherula sinensis* (Uvarov, 1925)
Acrididae	*Eremippus*	*Eremippus persicus* Uvarov, 19291
Acrididae		*Eremippus simplex* (Eversmann, 1859)
Acrididae		*Eremippus heimahoensis* Zheng&Hang, 1974
Acrididae	*Podismomorpha*	*Podismomorpha gibba* Lian&Zheng, 1984
Acrididae	*Squamopenna*	*Squamopenna gansuensis* Lian&Zheng, 1984
Acrididae	*Euchorthippus*	*Euchorthippus unicolor* (Ikonnikov, 1913)
Acrididae		*Euchorthippus weichowensis* Chang, 1937
Acrididae		*Euchorthippus flexucarinatus* Bi e Xia, 1987
Acrididae		*Euchorthippus cheui* Hsia, 1965
Acrididae		*Euchorthippus yungningensis* Cheng&Chiu, 1965
Acrididae	*Eclipophleps*	*Eclipophleps xizangensis* Liu, 1980
Acrididae	*Kangacris*	*Kangacris rufipes* Yin, 1983
Acrididae	*Macrokangacris*	*Macrokangacris luteoarmilla* Yin, 1983
Acrididae	*Kangacrisoides*	*Kangacrisoides jiangdaensis* Zheng, Dong, Bai&Xu, 2013
Acrididae	*Chorthippus*	*Chorthippus (Altichorthippus) intermedius* (Bey-Bienko, 1926)
Acrididae		*Chorthippus qingzangensis* Yin, 1984
Acrididae		*Chorthippus (Altichorthippus) latisulcus* (Zheng&He, 1995)
Acrididae		*Chorthippus (Chorthippus) dabanshanensis* Chen, Zeng&Bao, 2011
Acrididae		*Chorthippus (Chorthippus) qilianshanensis* Zheng&Xie, 2000
Acrididae		*Chorthippus(Altichorthippus) qinghaiensis* Wang&Zheng, 1992
Acrididae		*Chorthippus (Altichorthippus) jishishanensis* Zheng&Xie, 2000
Acrididae		*Chorthippus guideensis* Zheng&al, 2009
Acrididae		*Chorthippus (Altichorthippus) changtunensis* Yin, 1984
Acrididae		*Chorthippus flexivenus* (Liu, 1981)
Acrididae		*Chorthippus (Altichorthippus) chayuensis* Yin, 1984
Acrididae		*Chorthippus albonemus* Zheng&Tu, 1964
Acrididae		*Chorthippus intermedius* (Bey-Bienko, 1926)
Acrididae		*Chorthippus chinensis* (Tarbinsky, 1927)
Acrididae		*Chorthippus hsiai* Cheng&Tu, 1964
Acrididae		*Chorthippus longisonus* (Li&Yin, 1987)
Acrididae		*Chorthippus (Altichorthippus) markamensis* (Yin, 1984)
Acrididae		*Chorthippus biguttulus biguttulus* (Linnaeus, 1758)
Acrididae		*Chorthippus chapini* Chang, 1939
Acrididae		*Chorthippus grahami* Chang, 1937
Acrididae		*Chorthippus brevipterus* Yin, 1984
Acrididae		*Chorthippus fallax* (Zubovsky), 1899
Acrididae		*Chorthippus brunneus huabeiensis* Xia&Jin, 1982
Acrididae		*Chorthippus maerkangensis* (Zheng, 1980)
Acrididae		*Chorthippus xiangchengensis* (Liu, 1985)
Acrididae		*Chorthippus louguanensis* Zheng&Tu, 1964
Acrididae		*Chorthippus occidentalis* Xia&Jin, 1982
Acrididae		*Chorthippus squamopennis* Zheng, 1980
Acrididae		*Chorthippus dubius* (Zubovski, 1898)
Acrididae		*Chorthippus xiningensis* Zheng&Chen, 2001
Acrididae		*Chorthippus haibeiensis* Zheng&Chen, 2001
Acrididae		*Chorthippus helanshanensis* Zheng, 1999
Acrididae		*Chorthippus rufitibis* (Zheng, 1989)
Acrididae		*Chorthippus brunneus* (Thunbeng, 1815)
Acrididae		*Chorthippus xunhuaensis* Zheng&Xie, 2000
Acrididae		*Chorthippus leduensis* Zheng&Xin, 1999
Acrididae		*Chorthippus gongbuensis* Liang&Zheng, 1991
Acrididae		*Chorthippus mollis (*Charpentieri, 1825)
Acrididae		*Chorthippus amplintersitus* Liu, 1981
Acrididae		*Chorthippus rubensabdomenis* Liu, 1981
Acrididae		*Chorthippus hemipeterus* Uvarov, 1926
Acrididae		*Chorthippus flavabdomenis* Liu, 1981
Acrididae		*Chorthippus tibetanus* Uvarov, 1935
Acrididae		*Chorthippus separatanus* Liu, 1981
Acrididae		*Chorthippus aethalinus* (Zubovski, 1899)
Acrididae		*Chorthippus laifoveatus* Xia&Jin, 1982
Acrididae		*Chorthippus dichrous* (Eversmann, 1859)
Acrididae		*Chorthippus huchengensis* Xia&Jin, 1982
Acrididae		*Chorthippus daitongensis* Huo, 1994
Acrididae		*Chorthippus fusigeniculatus* Zheng&Men, 2008
Acrididae		*Chorthippus xueshanensis* Zheng&Mao, 1997
Acrididae		*Chorthippus deqinensis* Liu, 1984
Acrididae		*Chorthippus nemus* Liu, 1984
Acrididae		*Chorthippus yajiangensis* Zheng&Shi, 2007
Acrididae		*Chorthippus kangdingensis* Zheng&Shi, 2007
Acrididae		*Chorthippus neipopennis* Xia&Jin, 1982
Acrididae		*Chorthippus anomopterus* Liu, 1984
Acrididae	*Omocestus*	*Omocestus cuonaensis* Yin, 1984
Acrididae		*Omocestus gonggarensis* Zheng&Chen, 1995
Acrididae		*Omocestus haemorrhoidalis* (Charpentier, 1825)
Acrididae		*Omocestus avellaeusitibia* Zheng, Dong&Xu, 2013
Acrididae		*Omocestus petraeus* (Brisout de Barneville, 1856)
Acrididae		*Omocestus nyalamus* Xia, 1981
Acrididae		*Omocestus megaoculus* Yin, 1984
Acrididae		*Omocestus motuoensis* Yin, 1984
Acrididae		*Omocestus pinanensis* Zheng&Xie, 2001
Acrididae		*Omocestus qinghaihuensis* Zheng&Xie, 2001
Acrididae		*Omocestus hingstoni* Uvarov, 1925
Acrididae		*Omocestus enitor* Uvarov, 1924
Acrididae		*Omocestus laojunshanensis* Mao&Xu, 2004
Acrididae	*Nivisacris*	*Nivisacris zhongdianensis* Liu, 1984
Acrididae	*Saxetophilus*	*Saxetophilus qinghaiensis* Zheng, Chen&Li, 2012
Acrididae		*Saxetophilus petulans* Umnov, 1930
Acrididae		*Saxetophilus gansuensis* Wang, Zheng&Lian, 2006
Acrididae	*Oreoptygonotus*	*Oreoptygonotus chinghaiensis* (Zheng&Hang, 1974)
Acrididae		*Oreoptygonotus tibetanus* Tarbinsky, 1927
Acrididae		*Oreoptygonotus brachypterus* Yin, 1984
Acrididae		*Oreoptygonotus robustus* Yin, 1984
Acrididae		*Oreoptygonotus nigrofasciatus* Yin, 1979
Acrididae		*Oreoptygonotus belonocercus* Liu, 1981
Acrididae	*Ruganotus*	*Ruganotus rufipes* Yin, 1979
Acrididae	*Ptygonotus*	*Ptygonotus sichuanensis* Zheng, 1983
Acrididae		*Ptygonotus semenovi semenovi* Tarbinsky, 1927
Acrididae		*Ptygonotus tarbinskyi* Uvarov, 1930
Acrididae		*Ptygonotus gurneyi* Chang, 1937
Acrididae		*Ptygonotus chinghaiensis* Ying, 1974
Acrididae		*Ptygonotus hocashanensis* Cheng&Hang, 1974
Acrididae		*Ptygonotus brachypterus* Yin, 1974
Acrididae		*Ptygonotus gansuensis* Zheng&Chang, 1994
Acrididae		*Ptygonotus semenovi antennatus* Mistshenko, 1951
Acrididae		*Ptygonotus rangkouensis* Zheng&Xu, 2009
Acrididae		*Ptygonotus anurous* Zheng&Xu, 2009
Acrididae	*Asulconotoides*	*Asulconotoides sichuanensis* Liu, 1984
Acrididae		*Asulconotoides asulcata* (Zheng, 1988)
Acrididae		*Asulconotoides microptera* (Zheng, 1988)
Acrididae		*Asulconotus chinghaiensis* Ying, 1974
Acrididae		*Asulconotus jiuzhiensis* Yin, Zheng&Yin, 2012
Acrididae		*Asulconotus kozlovi* Mistshenko, 1981
Acrididae	*Asonus*	*Asonus brachypterus* (Ying, 1974)
Acrididae		*Asonus amplifurculus* Chen, Zheng&Zeng, 2010

## Appendix B. Checklist of Endemic Species in the Five Distribution Areas Identified by UPGMA: Himalayas (F8, F9), Bayan Har Mountains (D12, D13), Jinsha River Basin (E14, F14), Western Sichuan (E15, E16), and Hengduan Mountains (G14, G15)

**Endemic Distribution Area**	**Species Name**
Himalayas	*Bryodemella xizangensis* Yin, 1984
	*Bryodema pseudohyalinala* Zheng, Lin&Zhang, 2012
	*Bryodema kangmarensis* Zheng, Lin&Zhang, 2012
	*Eclipophleps xizangensis* Liu, 1980
	*Omocestus nyalamus* Xia, 1981
	*Omocestus megaoculus* Yin, 1984
	*Omocestus hingstoni* Uvarov, 1925
	*Hypernephia everesti* Uvarov, 1922
	*Dysanema irvinei* Uvarov, 1925
	*Dysanema magna* Zheng, Lin, Zhang&Zeng, 2014
	*Orinhippus tibetanus* Uvarov, 1921
	*Orinhippus trisulcus* Yin, 1984
Bayan Har Mountains	*Eokingdonella angqianensis* Chen&Zheng, 2009
	*Eokingdonella bayanharaensis* Huo, 1995
	*Kingdonella bicollina* Yin, 1984
	*Kingdonella qinghaiensis* Zheng, 1990
	*Kingdonella pienbaensis* Zheng, 1980
	*Kingdonella pictipes* Uvarov, 1935
	*Kingdonella kozlovi* Mistshenko, 1952
	*Bryodemella diamesum* (Bey-Bienko, 1930)
	*Bryodema miramae elegantulum* Bey-Bienko, 1930
	*Bryodema nigristria* Zheng&Chen, 2001
	*Ptygonotus sichuanensis* Zheng, 1983
	*Ptygonotus brachypterus* Yin, 1974
	*Asulconotus chinghaiensis* Ying, 1974
	*Asonus brachypterus* (Ying, 1974)
	*Asonus qinghaiensis* Liu, 1986
	*Pseudoasonus yushuensis* Yin, 1982
	*Pseudoasonus orthomarginis* Zheng, Chen&Lin, 2012
	*Aeropedelloides altissimus* Liu, 1981
	*Aeropedellus zadoensis* Yin, 1984
	*Aeropedellus prominemarginis* Zheng, 1981
Jinsha River Basin	*Pseudoasonus baiyuensis* Zheng, 1990
	*Gonista chayuensis* Yin, 1984
	*Tibetacris changtunensis* Chen, 1964
	*Chorthippus (Altichorthippus) changtunensis* Yin, 1984
	*Omocestus megaoculus* Yin, 1984
	*Sphingonotus caerulistriatus* Zheng&Ren, 2007
	*Chorthippus deqinensis* Liu, 1984
	*Anepipodisma punctata* Huang, 1984
	*Kingdonella bicollina* Yin, 1984
	*Mekongiana gregoryi* (Uvarov, 1925)
	*Chorthippus grahami* Chang, 1937
	*Kingdonella hanburyi* Uvarov, 1939
	*Omocestus avellaeusitibia* Zheng, Dong&Xu, 2013
	*Kingdonella nigrofemora* Yin, 1984
	*Kingdonella nigrotibia* Zheng, 1990
	*Leuconemacris hengduanshanensis* Zheng, 1988
	*Bryodemella diamesum* (Bey-Bienko, 1930)
	*Eokingdonella burrusitibia* Zheng, Dong&Xu, 2013
	*Kangacris rufipes* Yin, 1983
	*Macrokangacris luteoarmilla* Yin, 1983
	*Kangacrisoides jiangdaensis* Zheng, Dong, Bai&Xu, 2013
	*Chorthippus chapini* Chang, 1939
	*Chorthippus amplintersitus* Liu, 1981
	*Chorthippus nemus* Liu, 1984
	*Eokingdonella luhuoensis* Zheng&Shi, 2007
	*Chorthippus (Altichorthippus) markamensis* (Yin, 1984)
	*Aiolopus markamensis* Yin, 1984
	*Omocestus motuoensis* Yin, 1984
	*Bryodema miramae elegantulum* Bey-Bienko, 1930
	*Chorthippus(Altichorthippus) qinghaiensis* Wang&Zheng, 1992
	*Asulconotoides asulcata* (Zheng, 1988)
	*Leuconemacris xizangensis* (Yin, 1984)
	*Aeropus tibetanus* Uvarov, 1935
	*Parahilethera xizangensis* Zheng&Ren, 2007
	*Leuconemacris xiangchengensis* Zheng, 1988
	*Chorthippus xiangchengensis* (Liu, 1985)
	*Conophymacris xianggelilaensis* Niu&Zheng, 2009
	*Mekongiana xiangchengensis* Zheng, Huang&Zhou, 2008
	*Asulconotoides microptera* (Zheng, 1988)
	*Leuconemacris xinlongensis* Mao&Huang, 2023
	*Chorthippus anomopterus* Liu, 1984
	*Anaptygus yueliangwan* Mao, Ren&Ou, 2011
	*Leuconemacris longipennis* Zheng, 1988
	*Chorthippus longisonus* (Li&Yin, 1987)
	*Oreoptygonotus robustus* Yin, 1984
Western Sichuan	*Chorthippus (Altichorthippus) changtunensis* Yin, 1984
	*Leuconemacris breviptera* (Yin, 1983)
	*Ptygonotus gurneyi* Chang, 1937
	*Chorthippus grahami* Chang, 1937
	*Omocestus avellaeusitibia* Zheng, Dong&Xu, 2013
	*Conophymacris nigrofemora* Liang, 1993
	*Kingdonella nigrotibia* Zheng, 1990
	*Kangacris rufipes* Yin, 1983
	*Chorthippus occidentalis* Xia&Jin, 1982
	*Chorthippus chapini* Chang, 1939
	*Chorthippus kangdingensis* Zheng&Shi, 2007
	*Pseudoasonus kangdingensis* Yin, 1983
	*Eokingdonella cyanecuda* Zheng&Shi, 2009
	*Leuconemacris litangensis* (Yin, 1983)
	*Chorthippus squamopennis* Zheng, 1980
	*Chorthippus maerkangensis* (Zheng, 1980)
	*Eokingdonella luhuoensis* Zheng&Shi, 2007
	*Ptygonotus sichuanensis* Zheng, 1983
	*Ptygonotus anurous* Zheng&Xu, 2009
	*Leuconemacris xinlongensis* Mao&Huang, 2023
	*Transtympanacris xueshanensis* Lian&Zheng, 1985
	*Chorthippus yajiangensis* Zheng&Shi, 2007
	*Chorthippus neipopennis* Xia&Jin, 1982
	*Habrocnemis sinensis* Uvarov, 1930
Hengduan Mountains	*Yunnanites albomargina* Mao&Zheng, 1999
	*Pseudoasonus baiyuensis* Zheng, 1990
	*Caryanda bidentata* Zheng&Liang, 1985
	*Conophymacris nigrofemora* Liang, 1993
	*Omocestus laojunshanensis* Mao&Xu, 2004
	*Chorthippus nemus* Liu, 1984
	*Asulconotoides sichuanensis* Liu, 1984
	*Curvipennis wixiensis* Huang, 1984
	*Parapodisma astris* Huang, 2006
	*Leuconemacris xiangchengensis* Zheng, 1988
	*Conophymacris xianggelilaensis* Niu&Zheng, 2009
	*Asonus parvoculus* Xie & Liu, 1998
	*Chorthippus xueshanensis* Zheng&Mao, 1997
	*Altichorthippus yanyuanensis* Jin&Lin,1982
	*Anaptygus yulongensis* Wang, Zheng&Lian, 2005
	*Paramekongiella zhongdianensis* Huang, 1990
	*Anaptygus longipennis* Wang, Zheng&Lian, 2004
	*Yunnanites zhengi* Mao&Yang, 2003
	*Xiangelilacris zhongdianensis* Zheng, Huang&Zhou, 2008
	*Nivisacris zhongdianensis* Liu, 1984
	*Dianacris choui* Yin, 1983

## Appendix C. Checklist of Endemic Species in Three Endemic Distribution Areas Identified by the Parsimony Analysis of Endemism (PAE) Method

Jinsha River Basin (E14, F14), Hengduan Mountains (G14, G15), and Western Sichuan Hengduan Mountains Region (F15, E15, E16). The Jinsha River Basin (E14, F14) and Hengduan Mountains (G14, G15) were also identified by the Unweighted Pair Group Method with Arithmetic Mean (UPGMA) and are not repeated here.

**Endemic Distribution Area**	**Species Name**
Western Sichuan Hengduan Mountains Region	*Chorthippus (Altichorthippus) chayuensis* Yin, 1984
	*Chorthippus (Altichorthippus) changtunensis* Yin, 1984
	*Leuconemacris daochengensis* Zheng, 1988
	*Leuconemacris breviptera* (Yin, 1983)
	*Ptygonotus gurneyi* Chang, 1937
	*Chorthippus grahami* Chang, 1937
	*Omocestus avellaeusitibia* Zheng, Dong&Xu, 2013
	*Conophymacris nigrofemora* Liang, 1993
	*Kingdonella nigrotibia* Zheng, 1990
	*Chorthippus rubensabdomenis* Liu, 1981
	*Ochyracris rufotibialis* Zheng, 1991
	*Kangacris rufipes* Yin, 1983
	*Chorthippus occidentalis* Xia&Jin, 1982
	*Leuconemacris acuminata* Mao&Huang, 2023
	*Chorthippus chapini* Chang, 1939
	*Conophymacris jiulongensis* Zheng&Shi, 2009
	*Chorthippus kangdingensis* Zheng&Shi, 2007
	*Pseudoasonus kangdingensis* Yin, 1983
	*Eokingdonella cyanecuda* Zheng&Shi, 2009
	*Leuconemacris litangensis* (Yin, 1983)
	*Chorthippus squamopennis* Zheng, 1980
	*Eokingdonella luhuoensis* Zheng&Shi, 2007
	*Chorthippus maerkangensis* (Zheng, 1980)
	*Aiolopus markamensis* Yin, 1984
	*Leuconemacris muliensis* Mao&Huang 2023
	*Leuconemacris shalulishanensis* Zheng, 1988
	*Ptygonotus sichuanensis* Zheng, 1983
	*Asulconotoides sichuanensis* Liu, 1984
	*Ptygonotus anurous* Zheng&Xu, 2009
	*Leuconemacris xizangensis* (Yin, 1984)
	*Leuconemacris xiangchengensis* Zheng, 1988
	*Chorthippus xiangchengensis* (Liu, 1985)
	*Asonus parvoculus* Xie & Liu, 1998
	*Leuconemacris xinlongensis* Mao&Huang, 2023
	*Chorthippus xueshanensis* Zheng&Mao, 1997
	*Chorthippus yajiangensis* Zheng&Shi, 2007
	*Chorthippus neipopennis* Xia&Jin, 1982
	*Transtympanacris yajiangensis* Zheng, 1995
	*Chorthippus longisonus* (Li&Yin, 1987)
	*Habrocnemis sinensis* Uvarov, 1930

## Appendix D. Selection Results of Environmental Factors for Four Genera Within the Acridoidea

**Genus**	**Environmental Factors**
Locusta	Mean Diurnal Temperature Range (MDTR)
	Isotherm
	Seasonal Temperature
	Annual Temperature Range
	Annual Precipitation
	Precipitation in the Driest Month
	Seasonal Precipitation
	Precipitation in the Hottest Season
	Precipitation in the Coldest Season
	Elevation
	Wind Speed
	Solar Radiation
	Actual Evapotranspiration
	Potential Evapotranspiration
	Normalized Difference Vegetation Index (NDVI)
Leuconemacris	Isothermal Temperature
	Seasonal Temperature
	Annual Precipitation
	Precipitation in the Driest Month
	Precipitation in the Coldest Season
	Elevation
	Actual Evapotranspiration
	Solar Radiation
	Normalized Difference Vegetation Index (NDVI)
Kingdonella	Mean Diurnal Temperature Range (MDTR)
	Isotherm
	Precipitation in the Hottest Season
	Elevation
	Wind Speed
	Normalized Difference Vegetation Index (NDVI)
	Actual Evapotranspiration
Chorthippus	Mean Diurnal Temperature Range (MDTR)
	Isotherm
	Precipitation in the Hottest Season
	Elevation
	Wind Speed
	Normalized Difference Vegetation Index (NDVI)
	Actual Evapotranspiration

## Appendix E

**Table A1:** The suitable habitat area of *Locusta* in current, 2050s, and 2070s based on the prediction of the MaxEnt model.

	Non-Suitable Habitat (×10^4^ km^2^)	Suitable Habitat (×10^4^ km^2^)	Marginally Suitable Habitat (×10^4^ km^2^)	Moderately Suitable Habitat (×10^4^ km^2^)	Highly Suitable Habitat (×10^4^ km^2^)
**Current**	233.27	17.45	10.51	3.86	3.08
**Year 2050**	227.49	23.23	14.56	5.37	3.3
**Year 2070**	227.63	23.1	14.89	5.31	2.9

**Table A2:** Projected Changes in Suitable Habitat Area for *Locusta* Species by 2050 and 2070 Relative to the Current.

	Suitable Habitat (%)	Marginally Suitable Habitat (%)	Moderately Suitable Habitat (%)	Highly Suitable Habitat (%)
**Year 2050**	33.12	38.53	39.12	7.14
**Year 2070**	32.28	41.67	37.56	−5.84

**Table A3:** The suitable habitat area of *Leuconemacris* in current, 2050s, and 2070s based on the prediction of the MaxEnt model.

	Non-Suitable Habitat (×10^4^ km^2^)	Suitable Habitat (×10^4^ km^2^)	Marginally Suitable Habitat (×10^4^ km^2^)	Moderately Suitable Habitat (×10^4^ km^2^)	Highly Suitable Habitat (×10^4^ km^2^)
**Current**	239.28	11.48	5.65	3.15	2.68
**Year 2050**	239.44	11.29	5.51	3.99	1.79
**Year 2070**	239.88	10.86	5.43	3.67	1.76

**Table A4:** Projected Changes in Suitable Habitat Area for *Leuconemacris* Species by 2050 and 2070 Relative to the Current.

	Suitable Habitat (%)	Marginally Suitable Habitat (%)	Moderately Suitable Habitat (%)	Highly Suitable Habitat (%)
**Year 2050**	−1.66	−2.48	26.67	−33.21
**Year 2070**	−5.40	−3.89	16.51	−34.33

**Table A5:** The suitable habitat area of *Kingdonella* in current, 2050s, and 2070s based on the prediction of the MaxEnt model.

	Non-Suitable Habitat (×10^4^ km^2^)	Suitable Habitat (×10^4^ km^2^)	Marginally Suitable Habitat (×10^4^ km^2^)	Moderately Suitable Habitat (×10^4^ km^2^)	Highly Suitable Habitat (×10^4^ km^2^)
**Current**	229.4	21.34	12.84	5.29	3.21
**Year 2050**	224.8	25.92	16.37	6.41	3.14
**Year 2070**	225.71	25.03	15.65	5.95	3.43

**Table A6:** Projected Changes in Suitable Habitat Area for *Kingdonella* Species by 2050 and 2070 Relative to the Current.

	**Suitable Habitat** **(%)**	**Marginally Suitable Habitat** **(%)**	**Moderately Suitable Habitat** **(%)**	**Highly Suitable Habitat** **(%)**
**Year 2050**	21.46	27.49	21.17	−2.18
**Year 2070**	17.29	21.88	12.48	6.85

**Table A7:** The suitable habitat area of *Chorthippus* in current, 2050s, and 2070s based on the prediction of the MaxEnt model.

	Non-Suitable Habitat (×10^4^ km^2^)	Suitable Habitat (×10^4^ km^2^)	Marginally Suitable Habitat (×10^4^ km^2^)	Moderately Suitable Habitat (×10^4^ km^2^)	Highly Suitable Habitat (×10^4^ km^2^)
**Current**	216.31	34.41	20.21	8.91	5.29
**Year 2050**	211.86	38.86	22.1	10.86	5.9
**Year 2070**	212.13	38.6	21.24	11.26	6.1

**Table A8:** Projected Changes in Suitable Habitat Area for *Chorthippus* Species by 2050 and 2070 Relative to the Current.

	Suitable Habitat (%)	Marginally Suitable Habitat (%)	Moderately Suitable Habitat (%)	Highly Suitable Habitat (%)
**Year 2050**	12.93	9.35	21.89	11.53
**Year 2070**	12.18	5.10	26.37	15.31

## Appendix F. Species List of Acridoidea in the Qinghai–Tibet Plateau

**Species Name**	**Collection Site**	**Longitude**	**Latitude**
*Filchnerella sunanensis* Liu, 1982	Sunan Soap Alum Gully, Gansu	99.12	39.08
	Baidaban Grassland, Gansu	99.30	38.81
*Filchnerella zhengi* Huo, 1994	Ledu, Qinghai	102.40	36.48
*Filchnerella rufitibia* Yin, 1984	Huangliangchuan, Minhe County, Huangniangchuan, Qinghai	102.76	36.35
*Filchnerella kukunoris* Bei-Bienko, 1948	East Shore of Qinghai Lake, Gonghe County, Qinghai	100.44	37.09
*Filchnerella beicki* Ramme, 1931	East Shore of Qinghai Lake, Gonghe County, Qinghai	101.18	37.96
*Eoeotmethis longipennis* Zheng, 1985	Hongliu Gully, Sunan County, Gansu	99.24	39.10
*Beybienkia amicus* (Bey-Bienko, 1959)	Qilian Mountains, Gansu	101.18	37.96
*Aularches miliaris miliaris* (Linnaeus, 1758)	Mengda, Qinghai	102.69	35.82
*Mekongiella kingdoni* (Uvarov, 1937)	Linzhi Town, Linzhi City, Tibet	94.48	29.57
	Nanyi Gully, Milin County, Tibet	94.21	28.98
*Mekongiella pleurodilata* Yin, 1984	Cona, Tibet	91.96	28.00
	Lhünzhub, Tibet	91.61	31.44
*Mekongiella rufitibia* Yin, 1984	Cona, Tibet	91.96	28.00
*Mekongiella xizangensis* Yin, 1984	Cona, Tibet	91.96	28.00
	Nyingchi County, Tibet	93.24	29.89
*Mekongiella wardi* (Uvarov, 1937)	Quese Village, Nangqên County, Tibet	90.47	29.14
*Paramekongiella zhongdianensis* Huang, 1990	Hutiao Gorge, Shangri-La, Yunnan	100.11	27.21
*Mekongiana gregoryi* (Uvarov, 1925)	Xiangcheng County, Sichuan	99.80	28.93
	Deqin County, Yunnan	98.92	28.47
*Mekongiana xiangchengensis* Zheng, Huang&Zhou, 2008	Xiangcheng County, Sichuan	99.80	28.93
*Yunnanites coriacea* Uvarov, 1925	Xiangcheng County, Sichuan	99.80	28.93
	Lanping County, Yunnan	99.42	26.46
*Yunnanites zhengi* Mao&Yang, 2003	Shangri-La, Yunnan	99.74	27.84
	Ninglang County, Yunnan	100.85	27.29
*Yunnanites albomargina* Mao&Zheng, 1999	Lanping County, Yunnan	99.42	26.46
*Tagasta indica* Bolívar, 1905	Motuo, Tibet	95.33	29.33
*Atractomorpha melanostriga* Bi, 1981	Motuo, Tibet	95.33	29.33
	Cona, Tibet	91.96	28.00
	Xiachaiyu, Tibet	97.02	28.50
*Atractomorpha himalayica* Bolívar, 1905	Motuo, Tibet	95.33	29.33
	Xiachaiyu, Tibet	97.02	28.50
*Atractomorpha sinensis sinensis* Bolívar, 1905	Lanping County, Yunnan	99.42	26.46
	Gongshan County, Yunnan	98.67	27.74
	Fugong County, Yunnan	98.87	26.90
	Deqin County, Yunnan	98.92	28.47
	Shangri-La, Yunnan	99.74	27.84
	Weixi County, Yunnan	99.29	27.18
	Zhouqu, Gansu	104.37	33.79
	Guinan County, Qinghai	100.75	35.59
*Spathosternum prasiniferum xizangensis* Yin, 1982	Motuo, Tibet	95.33	29.33
	Beibeng, Motuo, Tibet	95.17	28.91
*Peripolus nepalensis* Uvarov, 1942	Zhangmu, Tibet	85.98	27.99
*Calliptamus barbarus barbarus* (Costa, 1836)	Guide County, Qinghai	101.43	36.04
	Jianzha, Qinghai	102.03	35.94
	Gandu Town, Hualong County, Qinghai	102.33	35.89
	Diebu County, Gansu	103.22	34.06
*Calliptamus abbreviatus* Ikonnikov, 1913	Xiahe, Gansu	102.52	35.20
	Zhuoni County, Gansu	103.51	34.59
	Qilian Mountain Bai Da Ban Grassland, Gansu	99.30	38.81
	Xining, Qinghai	101.78	36.62
	Gonghe County, Qinghai	100.62	36.28
	Guinan Mugetan, Qinghai	100.83	35.64
	Tongde County Kerbu Beach, Qinghai	100.48	35.34
	Guide County, Qinghai	101.43	36.04
	Jianzha, Qinghai	102.03	35.94
	Tongren County, Qinghai	102.02	35.52
	Tongren County, Qinghai	103.59	31.48
*Calliptamus italicus* (Linnaeus, 1758)	Guide County, Qinghai	101.43	36.04
	Xining, Qinghai	101.78	36.62
	Gangca County, Qinghai	100.14	37.32
	Zhiduo, Qinghai	95.62	33.84
	Gonghe County, Qinghai	100.62	36.28
*Xenocatantops brachycerus* (Willemse, 1932)	Chawalong Township, Tibet	98.46	28.48
	Mendu, Chayu County, Linzhi City, Tibet	97.03	28.75
	Chayu, Tibet	97.47	28.66
	Xiachayu, Chayu County, Tibet	96.99	28.50
	Diebu County, Gansu	103.22	34.06
	Zhouqu, Gansu	104.37	33.79
	Maqu County, Gansu	102.07	34.00
	Jinchuan County, Sichuan	102.06	31.48
	Tongren County, Qinghai	103.59	31.48
	Muli County, Sichuan	100.86	28.42
	Liziping Township, Muli County, Sichuan	100.29	27.97
	Shangri-La, Yunnan	99.74	27.84
	Weixi County, Yunnan	99.29	27.18
	Ninglang County, Yunnan	100.85	27.29
*Xenocatantops acanthraus* Zheng, Li&Wang, 2004	Motuo, Tibet	95.33	29.33
*Xenocatantops sauteri* (Ramme, 1941)	Motuo, Tibet	95.33	29.33
*Stenocatantops splendens* (Thunberg, 1815)	Xiachayu, Chayu County, Tibet	97.02	28.50
	Chayu, Tibet	97.40	28.83
*Stenocatantops mistshenkoi* Willemse, 1968	Jinchuan County, Sichuan	102.06	31.48
	Tongren County, Qinghai	103.59	31.48
*Catantops simlae* Dirsh, 1956	Jilong, Tibet	85.30	28.86
	Motuo, Tibet	95.33	29.33
*Catantops splendena* (Thunb.)	Motuo, Tibet	95.33	29.33
*Catantops pinguis pinguis* (Stål, 1860)	Shangri-La, Yunnan	99.74	27.84
	Muli County, Sichuan	100.86	28.42
	Xiachayu, Chayu County, Tibet	97.02	28.50
	Markam, Tibet	98.59	29.69
*Schistocerca gregaria* (Forskal, 1775)	Jilong, Tibet	85.30	28.86
	Dingri County, Tibet	87.12	28.66
	Dingjie County, Tibet	87.77	28.37
	Zhongba County, Tibet	84.03	29.77
	Nyalam, Tibet	85.98	28.16
*Patanga japonica* (Bolívar, 1898)	Chayu, Tibet	97.47	28.66
	Chawalong Township, Tibet	98.46	28.47
	Bomi, Tibet	95.77	29.86
	Motuo, Tibet	95.33	29.33
	Markam, Tibet	98.59	29.69
	Zhouqu, Gansu	104.37	33.79
	Jinchuan County, Sichuan	102.06	31.48
	Danba County, Sichuan	101.89	30.88
	Tongren County, Qinghai	103.59	31.48
	Kangding, Sichuan	101.95	30.16
	Xiangcheng County, Sichuan	99.80	28.93
	Ninglang County, Yunnan	100.85	27.29
	Deqin County, Yunnan	98.92	28.47
*Patanga humilis* Bi, 1986	Xiachayu, Chayu County, Tibet	97.02	28.50
	Motuo, Tibet	95.33	29.33
*Chondracris rosea brunneri* Uvarov, 1924	Motuo, Tibet108KM	95.27	29.30
	Motuo, Tibet	95.33	29.33
*Choroedocus robusia* (Servile, 1839)	Motuo, Tibet	95.33	29.33
*Shirakiacris yunkweiensis* (Chang, 1937)	Xiachayu, Chayu County, Tibet	97.02	28.50
	Zhouqu, Gansu	104.37	33.79
	Jinchuan County, Sichuan	102.06	31.48
	Tongren County, Qinghai	103.59	31.48
*Shirakiacris brachyptera* Zheng, 1983	Weixi County, Yunnan	99.29	27.18
*Eucoptacral moiuoensis* Yin, 1984	Motuo, Tibet	95.33	29.33
*Apalacris varicornis* Walker, 1870	Motuo, Tibet	95.33	29.33
*Apalacris xizangensis* Bi, 1984	Motuo, Tibet	95.33	29.33
	Beibeng, Motuo, Tibet	95.17	28.91
	Maniyung Village, Tibet	94.89	29.31
*Ecphanthacris mirabilis* Tinkham, 1940	Motuo, Tibet	95.33	29.33
*Assamacris longicercus* (Huang, 1981)	Motuo, Tibet108-80KM	95.27	29.30
	Motuo, Tibet	95.33	28.83
*Assamacris curticerca* (Huang, 1981)	Motuo, Tibet	95.33	28.83
	Hanmi Ani Bridge, Motuo, Tibet	95.29	29.33
*Promesosternus himalayicus* Yin, 1984	Motuo, Tibet	95.33	29.33
*Promesosternus vittatus* Yin, 1984	Motuo, Tibet	95.33	29.33
*Habrocnemis sinensis* Uvarov, 1930	Kangding, Sichuan	101.95	30.16
*Oxya agavisa agavisa* Tsai, 1931	Jiuzhaigou Valley, Sichuan	104.24	33.26
	Tongren County, Qinghai	103.59	31.48
*Oxya chinensis* (Thunberg, 1815)	Motuo, Tibet	95.33	29.33
*Oxya intricata* (Stål, 1861)	Motuo, Tibet	95.33	29.33
	Xiachayu, Chayu County, Tibet	97.02	28.50
	Diebu County, Gansu	103.22	34.06
	Luqu County, Gansu	102.49	34.39
*Oxya adentata* Willemse, 1925	Motuo, Tibet	95.33	29.33
	Chayu, Tibet	97.47	28.66
	Ledu District, Qinghai	102.40	36.48
*Oxya brachyptera* Zheng&Huo, 1992	Xining, Qinghai	101.78	36.62
*Oxya velox* (Fabricius, 1787)	Motuo, Tibet	95.33	29.33
*Oxya japonica* (Thunberg, 1824)	Motuo, Tibet	95.33	29.33
	Weixi County, Yunnan	99.29	27.18
*Caryanda gyirongensis* Huang, 1981	Xiaojilong, Tibet	85.33	28.40
*Caryanda bidentata* Zheng&Liang, 1985	Langfang Village, Lushui, Yunnan	98.60	26.11
*Parapodisma astris* Huang, 2006	Zhongdian Town, Shangri-La, Yunnan	99.79	27.58
	Baishuitai, Shangri-La, Yunnan	100.02	27.60
*Conophymacris chinensis* Willemse, 1933	Jade Dragon Snow Mountain, Lijiang, Yunnan	100.17	27.10
	Xiao Zhongdian, Yunnan	99.79	27.58
	Deqin County, Yunnan	98.92	28.47
	Kangding, Sichuan	102.33	30.25
*Conophymacris xianggelilaensis* Niu&Zheng, 2009	Xiao Zhongdian, Yunnan	99.79	27.58
	Zhongdian Town, Shangri-La, Yunnan	99.79	27.58
	Pushang, Shangri-La, Yunnan	99.74	28.15
	Jade Dragon Snow Mountain, Lijiang, Yunnan	100.17	27.09
*Conophymacris yunnanensis* Cheng, 1977	Wenhai Reservoir, Lijiang, Yunnan	100.16	26.98
*Conophymacris jiulongensis* Zheng&Shi, 2009	Wuxuhai Village, Jiulong County, Sichuan	101.41	29.15
	Hongba, Jiulong County, Sichuan	102.04	29.23
*Conophymacris cangshanensis* Zheng&Mao, 1996	Shangri-La, Yunnan	99.74	27.84
	Yunshanping, Lijiang, Yunnan	100.22	27.11
	Jade Dragon Snow Mountain, Lijiang, Yunnan	100.18	27.10
	Laojun Mountain, Jianchuan, Yunnan	99.59	26.56
*Conophymacris nigrofemora* Liang, 1993	Kangding, Sichuan	102.33	30.25
	Muli County, Sichuan	100.86	28.42
	Yunshanping, Lijiang, Yunnan	100.22	27.11
*Curvipennis wixiensis* Huang, 1984	Weixi County, Yunnan	99.29	27.18
	Lanping County, Yunnan	99.42	26.46
	Laojun Mountain, Jianchuan, Yunnan	99.59	26.56
*Pedopodisma emeiensis* (Yin, 1980)	Tongren County, Qinghai	103.59	31.48
*Pedopodisma abaensis* Yin, Zheng&Ye, 2012	Aba County, Sichuan	101.70	32.90
*Pseudozubovskia xizangensis* Zheng, Lin, Zhang&Zeng, 2014	Bomi, Tibet	95.77	29.86
*Sinopodisma zheng* Liang&Lin, 1995	Ninglang County, Yunnan	100.85	27.29
*Xiangelilacris zhongdianensis* Zheng, Huang&Zhou, 2008	Shangri-La, Yunnan	99.74	27.84
*Rectimargipodisma medogensis* Zheng, Li&Wang, 2004	Motuo, Tibet	95.33	29.33
*Indopodisma kingdoni* (Uvarov, 1927)	Chayu, Tibet	97.47	28.66
*Peseudoptygonotus alpinus*	Jinding Town, Lanping, Yunnan	99.41	26.43
*Fruhstorferiola omei* (Rehn&Rehn, 1939)	Tongren County, Qinghai	103.59	31.48
*Tylotropidius yunnanensis* Zheng&Liang, 1990	Shangri-La, Yunnan	99.74	27.84
*Rhinopodisma eminifrontus* (Huang, 1981)	Yigong, Bomi, Tibet	94.82	30.27
	Pailong, Nyingchi, Tibet	95.01	30.04
*Eokingdonella changtunica* Yin, 1984	Chamdo, Tibet	97.17	31.15
*Eokingdonella cyanecuda* Zheng&Shi, 2009	Yajiang, Sichuan	101.01	30.03
*Eokingdonella angqianensis* Chen&Zheng, 2009	Yushu, Qinghai	97.01	32.99
	Jiuzhi, Qinghai	101.48	33.43
	Zhiduo, Qinghai	95.62	33.84
	Qumarlêb, Qinghai	95.80	34.13
	Nagqu, Qinghai	96.49	32.21
	Zadoi, Qinghai	95.30	32.89
	Gade, Qinghai	99.90	33.97
	Maqin, Qinghai	100.24	34.48
	Tongde, Qinghai	100.58	35.25
	Niangra Township, Qinghai	96.84	32.04
*Eokingdonella gongbugyanda* Zheng, Lin, Zhang&Zeng, 2014	Gongbujiangda, Tibet	93.24	29.89
*Eokingdonella gentiana* (Uvarvo, 1939)	Basu, Tibet	96.92	30.05
*Eokingdonella tibetana* (Mistshenko, 1952)	Basu, Tibet	96.92	30.05
*Eokingdonella burrusitibia* Zheng, Dong&Xu, 2013	Jiangda County, Tibet	98.14	31.44
*Eokingdonella kaulbacki* (Uvarvo, 1939)	Zuogong, Tibet	97.84	29.67
*Eokingdonella bayanharaensis* Huo, 1995	Yushu, Qinghai	97.01	32.99
	Jiuzhi, Qinghai	101.48	33.43
	Zhiduo, Qinghai	95.62	33.84
	Qumarlêb, Qinghai	95.80	34.13
	Nagqu, Qinghai	96.49	32.21
	Zadoi, Qinghai	95.30	32.89
	Gade, Qinghai	99.90	33.97
	Maqin, Qinghai	100.24	34.48
	Tongde, Qinghai	100.58	35.25
*Eokingdonella luhuoensis* Zheng&Shi, 2007	Ganzi County, Sichuan	99.99	31.62
	Luhuo, Sichuan	100.68	31.39
*Kingdonella bicollina* Yin, 1984	Racecourse, Yushu, Qinghai	96.96	33.00
	Nagqu, Qinghai	96.48	32.19
	Baizha township, Nagqu, Qinghai	96.52	32.07
	Duocai, Zhiduo, Qinghai	95.42	33.81
	Riwoqe, Tibet	96.60	31.21
	Dzumbala, Markam, Tibet	98.62	29.72
	Xiaqu Town, Biru, Tibet	92.83	31.81
	Kagong Township, Jiangda County, Tibet	98.14	31.44
	Jiangda County, Tibet	98.23	31.50
	Kagong Township, Jiangda County, Tibet	97.99	31.29
	Ganzi County, Sichuan	99.99	31.62
*Kingdonella qinghaiensis* Zheng, 1990	Duocai, Zhiduo, Qinghai	95.42	33.81
	Baizha township, Nagqu, Qinghai	96.52	32.07
	Racecourse, Yushu, Qinghai	96.96	33.00
	Tongde, Qinghai	100.58	35.25
	Jiuzhi, Qinghai	101.48	33.43
*Kingdonella pienbaensis* Zheng, 1980	Xiaqu Town, Biru, Tibet	92.83	31.81
	Kagong Township, Jiangda County, Tibet	97.99	31.29
	Bianba, Tibet	94.71	30.93
	Balila Mountain, Bianba, Tibet	95.27	30.79
	Demula Mountain, Chayu, Qinghai	97.03	29.32
	Chabatang, Chayu, Qinghai	97.22	32.83
	Shangbatang, Chayu, Qinghai	97.07	32.85
	Yushu, Qinghai	97.01	32.99
	Bulang, Chayu, Qinghai	96.77	33.35
	Longbao, Chayu, Qinghai	96.42	33.26
	Gelong, Chayu, Qinghai	95.56	32.64
	Xiao Sumang, Chayu, Qinghai	97.25	32.33
	Duocai, Zhiduo, Qinghai	95.42	33.81
*Kingdonella nigrotibia* Zheng, 1990	Maharong Gully, Baiyu, Sichuan	98.87	31.17
	Honglong Town, Yajiang, Sichuan	100.52	30.11
*Kingdonella magna* Yin, 1984	Dari County, Qinghai	99.65	33.75
*Kingdonella nigrofemora* Yin, 1984	Jiangda County, Tibet	98.10	31.80
	Jiuzhi, Qinghai	101.48	33.43
*Kingdonella hanburyi* Uvarov, 1939	Zuogong, Tibet	97.84	29.67
	Basu, Tibet	96.92	30.05
	Ganzi County, Sichuan	99.99	31.62
*Kingdonella parvula* Yin, 1984	Riwoqe, Tibet	96.60	31.21
*Kingdonella longiconica* Yin, 1984	Mount Kaji La, Tibet	96.40	31.60
*Kingdonella pictipes* Uvarov, 1935	Ranwu Town, Tibet	96.75	29.51
	Riwoqe, Tibet	96.60	31.21
	Chabatang, Chayu, Qinghai	97.22	32.83
	Shangbatang, Chayu, Qinghai	97.07	32.85
	Yushu, Qinghai	97.01	32.99
	Longbao, Chayu, Qinghai	96.42	33.26
	Xiao Sumang, Chayu, Qinghai	97.25	32.33
	Bulang, Chayu, Qinghai	96.77	33.35
*Kingdonella kozlovi* Mistshenko, 1952	Jiuzhi, Qinghai	101.48	33.43
	Yushu, Qinghai	97.01	32.99
	Chamdo, Tibet	97.17	31.15
*Kingdonella conica* Yin, 1984	Riwoqe, Tibet	96.60	31.21
*Kingdonella afurcula* Yin, 1984	Riwoqe, Tibet	96.60	31.21
*Kingdonella saxicola* Uvarvo, 1939	Basu, Tibet	96.92	30.05
*Kingdonella rivuna* Huang, 1981	Chaya Jitang, Tibet	97.35	30.74
*Kingdonella wardi* Uvarvo, 1933	Chayu, Tibet	97.47	28.66
*Kingdonella conica* Yin, 1984	Mount Kaji La, Tibet	96.40	31.60
*Anepipodisma punctata* Huang, 1984	Markam, Tibet	98.59	29.69
	Chayu, Tibet	97.47	28.66
	Deqin County, Yunnan	98.92	28.47
*Conophymopsis linguspinus* Huang, 1983	Tashkurgan Tajik Autonomous County, Xinjiang	75.23	37.77
	Akto County, Xinjiang	75.37	38.84
*Conophymopsis labrispinus* Huang, 1983	Yecheng County, Xinjiang	76.70	37.22
*Dericorys annulata roseipennis* (Redtenbacher, 1889)	Xiahe, Gansu	102.52	35.20
*Leptopternis gracilis* (Eversmann, 1848)	Qilian Mountains, Jiayuguan, Gansu	98.29	39.32
*Heteropternis respondens* (Walker, 1859)	Markam, Tibet	98.59	29.69
	Jinchuan County, Sichuan	102.06	31.48
	Deqin County, Yunnan	98.92	28.47
*Heteropternis robusta* Bey-Bienko, 1951	Markam, Tibet	98.59	29.69
	Xiangcheng County, Sichuan	99.80	28.93
	Kangding, Sichuan	101.95	30.16
	Luding County, Sichuan	102.23	29.92
	Yanyuan County, Sichuan	101.39	27.69
	Lanping County, Yunnan	99.42	26.46
	Shangri-La, Yunnan	99.74	27.84
	Deqin County, Yunnan	98.92	28.47
	Ninglang County, Yunnan	100.85	27.29
*Heteropternis motuoensis* Yin, 1984	Motuo, Tibet	95.33	29.33
*Heteropternis qinghaiensis* Wang&Zheng, 1992	Qumarlêb, Qinghai	95.80	34.13
*Heteropternis micronus* Huang, 1981	Xiachayu, Chayu County, Tibet	97.02	28.50
	Songgula Ga, Tibet	97.24	29.16
	Shangri-La, Yunnan	99.74	27.84
*Pusana chayuensis* (Yin, 1984)	Chayu, Tibet	97.47	28.66
*Mecostethus grossus* (Linnaeus, 1758)	Qilian County, Qinghai	100.25	38.18
	Guinan County, Qinghai	100.75	35.59
	Kangding, Sichuan	101.95	30.16
	Ruoergai County, Sichuan	102.96	33.58
*Ochyracris rufotibialis* Zheng, 1991	Jinping Mountain, Mianyang County, Sichuan	101.82	28.46
*Epacromius coerulipes* (Ivanov, 1888)	Guinan County, Qinghai	100.75	35.59
	Daotang River, Gonghe County, Qinghai	100.97	36.40
	Guide County, Qinghai	101.43	36.04
	Xining, Qinghai	101.78	36.62
	Ledu District, Qinghai	102.40	36.48
	Ping’an County, Qinghai	102.11	36.50
	Xunhua County, Qinghai	102.49	35.85
*Epacromius tergestinus tergestinus* (Megerle von Mühlfeld, 1825)	Wutumeiren Township, Geermu, Qinghai	93.16	36.91
	Tashkurgan Tajik Autonomous County, Xinjiang	75.23	37.77
	Xiachayu, Chayu County, Tibet	97.02	28.50
	Chayu, Tibet	97.40	28.83
*Epacromius tergestinus extimus* Bey-Bienko, 1951	Ledu District, Qinghai	102.40	36.48
	Geermu, Qinghai	94.90	36.40
	Dachaidan, Qinghai	95.36	37.85
	Guinan County, Qinghai	100.75	35.59
	Xining, Qinghai	101.78	36.62
	Muli Tibetan Autonomous County, Sichuan	100.86	28.42
*Aiolopus thalassinus* (Fabricius, 1798)	Deqin County, Yunnan	98.92	28.47
*Aiolopus tamulus* (Fabricius, 1798)	Luhuo, Sichuan	100.68	31.39
	Chawalong Township, Tibet	98.46	28.47
	Chayu, Tibet	97.47	28.66
*Aiolopus markamensis* Yin, 1984	Markam, Tibet	98.59	29.69
	Xiachayu, Chayu County, Tibet	97.02	28.50
	Muli County, Sichuan	100.86	28.42
	Derong County, Sichuan	99.29	28.72
*Oedaleus infernalis* Saussure, 1884	Xiahe, Gansu	102.52	35.20
	Qilian Shan Grassland, Bai Da Ban Prairie, Gansu	99.30	38.81
	Daotang River, Gonghe County, Qinghai	100.97	36.40
	Jianzha, Qinghai	102.03	35.94
	Hualong Hui Autonomous County, Qinghai	102.26	36.09
	Xunhua County, Qinghai	102.49	35.85
	Guide County, Qinghai	101.43	36.04
	Tongren County, Qinghai	102.02	35.52
	Xining, Qinghai	101.78	36.62
	Datong Hui and Tu Autonomous County, Qinghai	101.69	36.93
	Ping’an County, Qinghai	102.11	36.50
	Huzhu Tuzu Zizhixian, Qinghai	101.96	36.84
	Menyuan Hui Autonomous County, Qinghai	101.61	37.39
*Oedaleus asiaaicus* Bey-Bienko, 1941	Xining, Qinghai	101.78	36.62
	Gonghe County, Qinghai	100.97	36.40
	Tongde, Qinghai	100.58	35.25
	Guide County, Qinghai	101.43	36.04
	Jianzha, Qinghai	102.03	35.94
	Guinan County, Qinghai	100.75	35.59
	Hualong Hui Autonomous County, Qinghai	102.26	36.09
	Tongren County, Qinghai	102.02	35.52
	Datong Hui and Tu Autonomous County, Qinghai	101.69	36.93
	Ping’an County, Qinghai	102.11	36.50
	Huzhu Tuzu Zizhixian, Qinghai	101.96	36.84
	Menyuan Hui Autonomous County, Qinghai	101.61	37.39
	Diebu County, Gansu	103.22	34.06
	Xiahe, Gansu	102.44	35.06
	Jinchuan County, Sichuan	102.06	31.48
	Daocheng County, Sichuan	100.29	28.72
	Tongren County, Qinghai	103.59	31.48
	Maerkang County, Sichuan	102.20	31.91
*Oedaleus manjius* Chang, 1939	Muli County, Sichuan	100.86	28.42
	Ganzi County, Sichuan	99.99	31.62
	Tongren County, Qinghai	103.59	31.48
*Acrotylus longipes subfasciatus* Bey-Bienko, 1984	Zhangmu, Tibet	85.98	27.99
*Aurilobulus splendens* Yin, 1979	Zanda Xiangquan River Collection Site, Tibet	80.30	31.11
*Cyanicaudata annulicornea* Yin, 1979	Zanda Xiangquan River Collection Site, Tibet	80.30	31.11
*Celes skalozubovi* Adelung, 1906	Jianzha, Qinghai	102.03	35.94
	Tongde, Qinghai	100.58	35.25
	Guinan County, Qinghai	100.75	35.59
	Lintan County, Gansu	103.53	34.79
*Celes akitanus* (Shiraki, 1910)	Jianzha, Qinghai	102.03	35.94
*Sphingonotus kueideensis* Yin, 1984	Guide County, Qinghai	101.43	36.04
*Sphingonotus qinghaiensis* Yin, 1984	Jianzha, Qinghai	102.03	35.94
*Sphingonotus zhengi* Huo, 1994	Ledu District, Qinghai	102.40	36.48
*Sphingonotus yunnaneus* Uvarov,1925	Markam, Tibet	98.59	29.69
	Xiangcheng County, Sichuan	99.80	28.93
	Deiqen Moon Bay, Yunnan	99.31	28.20
	Shangri-La, Yunnan	99.74	27.84
*Sphingonotus tipicus* Zheng&Hang, 1974	Gonghe Tiebujia, Qinghai	99.58	37.03
*Sphingonotus halophilus* Bey-Bienko, 1929	Dachaidan Administrative Committee, Haixi Tibetan Autonomous Prefecture, Qinghai	95.36	37.86
*Sphingonotus tzaidamicus* Mistshenko,1937	Delhi City, Qinghai	97.36	37.37
	Dachaidan, Qinghai	95.36	37.85
	Geermu, Qinghai	94.90	36.40
	Dulan County, Qinghai	98.10	36.30
	Tanggula, Qinghai	92.44	34.21
*Sphingonotus caerulistriatus* Zheng&Ren,2007	Yanjing, Markam, Tibet	98.61	29.61
*Sphingonotus zadaensis* Huang, 1981	Zanda Mayang, Tibet	78.96	31.79
*Sphingonotus longipennis* Saussure, 1884	Chayu, Tibet	97.47	28.66
	Xiachaiyu, Tibet	97.02	28.50
*Sphingonotus mengolicus* Saussure, 1884	Diebu County, Gansu	103.22	34.06
*Sphingonotus obscuratus* latissimus Uvarvo, 1925	Tashkurgan Tajik Autonomous County, Xinjiang	75.23	37.77
*Sphingonotus elegans* Mistshenko, 1937	Tashkurgan Tajik Autonomous County, Xinjiang	75.23	37.77
*Sphingonotus turcmenus* Bey-Bienko, 1951	Dachaidan, Qinghai	95.36	37.85
*Tibetacris changtunensis* Chen, 1964	Chamdo, Tibet	97.17	31.15
	Chayagang, Tibet	97.35	30.74
	Yanjing, Markam, Tibet	98.61	29.61
*Trilophidia annulata* (Thunberg, 1815)	Motuo, Tibet	95.33	29.33
	Qilian Moutain, Qinghai	100.34	38.30
	Diebu County, Gansu	103.22	34.06
	Zhouqu, Gansu	104.37	33.79
	Xiahe, Gansu	102.44	35.06
	Muli County, Sichuan	100.86	28.42
	Weixi County, Yunnan	99.29	27.18
	Lanping County, Yunnan	99.42	26.46
*Parahilethera xizangensis* Zheng&Ren, 2007	Chamdo, Tibet	97.17	31.15
	Yanjing, Markam, Tibet	98.61	29.61
*Gastrimargus africanus africanus* (Saussure, 1888)	Zhangmu, Tibet	85.98	27.99
	Motuo, Tibet	95.33	29.33
	Jinchuan County, Sichuan	102.06	31.48
	Luding County, Sichuan	102.23	29.92
	Weixi County, Yunnan	99.29	27.18
	Laojun Mountain, Jianchuan, Yunnan	99.59	26.56
	Shangri-La, Yunnan	99.74	27.84
*Gastrimargus nubilis* Uvarov, 1925	Chamdo, Tibet	97.17	31.15
	Markam, Tibet	98.59	29.69
	Maerkang County, Sichuan	102.20	31.91
	Jinchuan County, Sichuan	102.06	31.48
	Lixian County, Sichuan	103.16	31.44
	Batang County, Sichuan	99.11	30.01
	Kangding, Sichuan	102.33	30.50
	Daocheng County, Sichuan	100.29	28.72
	Ganzi County, Sichuan	99.99	31.62
	Shangri-La, Yunnan	99.74	27.84
	Deqin County, Yunnan	98.92	28.47
	Ninglang County, Yunnan	100.85	27.29
	Weixi County, Yunnan	99.29	27.18
	Lanping County, Yunnan	99.42	26.46
*Gastrimargus marmoratus* (Thunberg, 1815)	Jinchuan County, Sichuan	102.06	31.48
	Luding County, Sichuan	102.23	29.92
	Jinsha River, Markam, Tibet	99.01	29.62
*Gastrimargus ommatidius* Huang, 1981	Chayab, Tibet	97.62	30.61
*Gastrimargus africanus parvulus* Sjöstedt, 1928	Deiqen Moon Bay, Yunnan	99.31	28.20
*Locusta migratoria migratorioides* (Reiche & Fairmaire, 1849)	Qingduo Township, Bomi, Tibet	95.60	30.06
	Dongga Township, Shigatse, Tibet	88.83	29.36
	Qubuxiong, Shigatse, Tibet	88.87	29.18
	Tongmai, Bomi, Tibet	95.08	30.10
	Guxiang, Bomi, Tibet	95.50	29.91
	Dongjiu, Nyingchi, Tibet	94.80	29.94
	Bayi Town North, Nyingchi, Tibet	94.36	29.65
	Dingri, Shigatse, Tibet	88.85	29.00
	Gongbujiangda, Tibet	93.24	29.89
	Linzhi Town, Linzhi City, Tibet	94.48	29.57
	Baiba, Nyingchi, Tibet	94.30	29.72
	Yigong, Bomi, Tibet	95.03	30.15
	Milin County, Tibet	94.22	29.21
	Pulan County, Tibet	81.18	30.29
	Sangri County, Tibet	92.02	29.26
	Zhanang, Tibet	91.34	29.24
	Zada Naga, Tibet	79.74	31.54
	Shigatse, Tibet	88.88	29.27
	Zhada Xiangzi, Tibet	79.63	31.83
	Rutog County, Tibet	79.73	33.38
	Gar County, Tibet	80.09	32.49
	Namling County, Tibet	89.10	29.69
	Gar County, Tibet	89.73	28.86
	Sakya County, Tibet	88.02	28.90
	Tongmen Township, Tibet	88.20	29.42
	Geji County, Tibet	81.14	32.40
	Gongjue, Tibet	98.27	30.86
	Zuogong, Tibet	97.84	29.67
	Yanjing, Tibet	98.61	29.61
	Jilong, Tibet	85.30	28.86
	Motuo, Tibet	95.33	29.33
	Linzho County, Tibet	91.26	29.90
	Lhasa, Tibet	91.17	29.66
	Chayu, Tibet	97.47	28.66
	Chayu, Tibet Shangcaoyu	96.78	28.72
	Nedong County, Zetang, Tibet	91.76	29.23
	Lazi County, Tibet	87.64	29.08
	Bai Zha Township, Nagqu, Qinghai	96.52	32.07
	Nagqu, Qinghai	96.48	32.19
	Yushu, Qinghai	97.01	32.99
	Luhuo, Sichuan	100.68	31.39
	Yimu Township, Luhuo, Sichuan	100.77	31.29
	Luoshu, Shiqiu County, Sichuan	98.00	32.47
	Gengqing Town, Dege County, Sichuan	98.58	31.82
	Ganzi County, Sichuan	99.99	31.62
	Zhengke Township, Shiqiu County, Sichuan	97.83	32.52
	Litang County, Ganzi, Sichuan	100.27	30.00
	Zangba Township, Litang County, Sichuan	100.44	29.65
	Xiangcheng County, Sichuan	99.80	28.93
	Bamei, Daofu, Sichuan	101.48	30.48
	Xiahe, Gansu	102.52	35.20
	Diebu County, Gansu	103.22	34.06
	Lintan County, Gansu	103.53	34.79
	Jinchuan County, Sichuan	102.06	31.48
	Ganzi County, Sichuan	99.99	31.62
	Muli County, Sichuan	100.86	28.42
	Shangri-La, Yunnan	99.74	27.84
	Deqin County, Yunnan	98.92	28.47
	Weixi County, Yunnan	99.29	27.18
	Ninglang County, Yunnan	100.85	27.29
*Locusta migratoria migratoria* (Linnaeus, 1758)	Xining, Qinghai	101.78	36.62
	Guinan County, Qinghai	100.75	35.59
*Pternoscirta saueri* (Karny, 1915)	Motuo, Tibet	95.33	29.33
*Pternoscirta pulchripes* Uvarov, 1925	Weixi County, Yunnan	99.29	27.18
	Lanping County, Yunnan	99.30	26.59
*Pternoscirta longipennis* Xia, 1981	Xiaojilong, Jilong, Tibet	85.33	28.40
*Bryodemella xizangensis* Yin, 1984	Shigatse, Tibet	88.88	29.27
	Chamdo, Tibet	96.35	30.51
	Dingri County, Tibet	86.98	28.47
	Saga, Tibet	85.27	29.38
	Sakya, Tibet	88.02	28.90
*Bryodemella tuberculatum dilutum* (Stoll, 1813)	Xiahe, Gansu	102.52	35.20
	Luqu County, Gansu	102.49	34.39
	Heiyatou, Qilian Mountains, Sunan, Gansu	100.00	38.50
	Dongliugou, Sunan, Gansu	99.64	38.77
	Zaofangou, Sunan, Gansu	99.12	39.08
	Nedong County, Zetang, Tibet	91.76	29.23
	Namtso, Damxung, Tibet	90.64	30.66
	Quse, Nangqên County, Tibet	90.47	29.14
	Yamdrok Lake, Nangqên County, Tibet	90.62	29.19
	Bairang Monastery, Tibet	89.23	29.14
	Lumbu Monastery, Jiangzi County, Tibet	89.73	28.86
	Yamdrok Lake, Tibet	90.71	28.92
	Jiangtang Village, Sangri County, Tibet	91.98	29.25
	Rongbu Town, Sog County, Tibet	94.65	31.55
	Duina Township, Yatung County, Tibet	89.23	27.98
	Heika Mountain, Xinghai County, Qinghai	100.05	35.78
	Daotang River, Gonghe County, Qinghai	100.97	36.40
	Qilian County, Qinghai	100.25	38.18
	Tongde, Qinghai	100.58	35.25
	Jianzha, Qinghai	101.88	35.93
	Ruoergai County, Sichuan Erlang Mountain	102.79	34.03
	Ruoergai County, Sichuan	102.96	33.58
	Maerkang County, Sichuan	102.20	31.91
	Hongyuan County, Sichuan	102.54	32.79
	Jinchuan County, Sichuan	102.06	31.48
	Ganzi County, Sichuan	99.99	31.62
*Bryodemella diamesum* (Bey-Bienko, 1930)	Batang, Yushu, Qinghai	97.15	32.83
	Racecourse, Yushu, Qinghai	96.96	33.00
	Zadoi, Qinghai	95.30	32.89
	Jiangda County, Tibet	98.22	31.50
	Riwoqe, Tibet	96.60	31.21
	Chamdo, Tibet	97.17	31.15
*Bryodemella (Bryodemella) rufifemura* Zhang, Zhi&Zhang, 2018	Qushe Village, Lhazê County, Tibet	90.47	29.14
*Bryodemella (Bryodemella) nigrifemura* Yin&Wang, 2005	Nedong County, Zetang, Tibet	91.76	29.23
	Lhasa, Tibet	91.13	29.66
	Lhazê County, Shannan, Tibet	90.40	28.97
	Yamdrok Lake, Tibet	90.71	28.92
*Bryodemella holdereri holdereri* (Krauss, 1901)	Xiahe, Gansu	102.52	35.20
	Dongliugou, Gansu	99.64	38.77
	Xining, Qinghai	101.78	36.62
	Daotang River, Gonghe County, Qinghai	100.97	36.40
	Tongde, Qinghai	100.58	35.25
	Jianzha, Qinghai	102.03	35.94
	Xinghai, Qinghai	99.99	35.59
	Guinan County, Qinghai	100.75	35.59
	Guide County, Qinghai	101.43	36.04
	Tongren County, Qinghai	102.02	35.52
	Datong Hui and Tu Autonomous County, Qinghai	101.69	36.93
	Ping’an County, Qinghai	102.11	36.50
	Huzhu Tuzu Zizhixian, Qinghai	101.96	36.84
	Menyuan Hui Autonomous County, Qinghai	101.61	37.39
*Bryodema luctuosum luctuosum* (Stoll, 1813)	Rutog County, Tibet	79.73	33.38
	Amdo, Tibet	91.33	32.33
	Gar County, Tibet	80.09	32.49
	Pulan County, Tibet	81.18	30.29
	Damxung County, Tibet	91.10	30.48
	Yangbajing, Tibet	90.54	30.10
	Namtso, Damxung, Tibet	90.64	30.66
	Nagqu City, Tibet	92.05	31.48
	Nima County, Heping Township, Tibet	88.63	32.13
	Shenzha County, Tibet	88.71	30.93
	Biru County, Datang Township, Tibet	93.11	31.53
	Bange County, Beila Town, Tibet	90.83	31.40
	Bange County, Mendang Township, Tibet	89.75	31.42
	Gegye County, Wenbu Dang Sang Township, Tibet	82.90	32.39
	Geji County, Salt Lake Township, Tibet	82.46	32.53
	Amdo, Tibet	91.68	32.26
	Nagqu Bange County, Tibet	90.01	31.39
	Duina Township, Yatung County, Tibet	89.23	27.98
	Gaize County, Dongcuo Township, Tibet	84.88	32.10
	Lhasa Linzhou, Tibet	91.26	29.90
	Lhasa, Tibet	91.17	29.66
	Pulan County, Mapam Yumco, Tibet	81.47	30.66
	Pulan County, Huo’er Township, Tibet	81.61	30.77
	Gaize County, Tibet	84.05	32.30
	Zanda County, Xiangzi Township, Tibet	79.63	31.83
	Cuoqin County, Tibet	85.15	31.02
	Cuoqin County, Xiabula Mountain, Tibet	85.04	31.16
	Lhazê County, Tibet	90.40	28.97
	Maqin, Qinghai	100.24	34.48
	Guinan County, Qinghai	100.87	35.73
	Racecourse, Yushu, Qinghai	96.96	33.00
	Zhiduo, Qinghai	95.61	33.87
	Duocai, Zhiduo, Qinghai	95.42	33.81
	Batang, Yushu, Qinghai	97.15	32.83
	Madoi County, Golog Tibetan Autonomous Prefecture, Qinghai	98.21	34.92
	Nian Di Township, Gonghe County, Qinghai	100.39	36.38
	Gonghe Tiebujia, Qinghai	99.58	37.03
	Hei Ma He Township, Gonghe County, Qinghai	99.78	36.73
	Ziketan, Xinghai County, Qinghai	99.98	35.59
	Batan, Qinghai	101.31	35.32
	Xining, Qinghai	101.78	36.62
	Jianzha, Qinghai	102.03	35.94
	Qilian County, Qinghai	100.25	38.18
	Tongren County, Qinghai	102.02	35.52
	Tianjun County, Qinghai	99.02	37.30
	Madoi County, Qinghai	98.21	34.91
	Qinghai Lake	100.35	36.75
	Altun Mountain Range, Xinjiang	90.16	38.39
	Shaziquan, Xinjiang	88.48	38.12
	Xiahe, Gansu	102.44	35.06
*Bryodema miramae miramae* Bey-Bienko, 1930	Dongliugou, Sunan, Gansu	99.64	38.77
	Heiyatou, Qilian Mountains, Sunan, Gansu	100.00	38.50
	Gonghe County, Qinghai	100.62	36.28
	Qilian County, Qinghai	100.25	38.18
	Xining, Qinghai	101.78	36.62
	Datong Hui and Tu Autonomous County, Qinghai	101.69	36.93
	Ping’an County, Qinghai	102.11	36.50
	Huzhu Tuzu Zizhixian, Qinghai	101.96	36.84
	Menyuan Hui Autonomous County, Qinghai	101.61	37.39
*Bryodema uvarovi* (Bey-Bienko, 1930)	Dongliugou, Sunan, Gansu	99.64	38.77
	Jianzha, Qinghai	102.03	35.94
	Tongren County, Qinghai	102.02	35.52
	Guide County, Qinghai	101.43	36.04
	Tongde, Qinghai	100.58	35.25
*Bryodema miramae elegantulum* Bey-Bienko, 1930	Yushu, Qinghai	97.01	32.99
	Qilian County, Qinghai	100.25	38.18
	Gongjue, Tibet	98.27	30.86
	Chamdo, Tibet	97.17	31.15
*Bryodema luctuosum inda* Saussure, 1884	Ziketan, Xinghai County, Qinghai	99.98	35.59
	Taga, Tibet	91.79	28.38
	Shannan City, Tibet	91.89	28.77
	Linzhi, Tibet	95.46	29.39
	Cona, Tibet	92.17	28.09
	Motuo, Tibet	95.00	29.33
	Chayu, Tibet	97.40	28.83
	Lhünzhub County, Tibet	92.79	28.45
	Himalayas, Tibet	85.17	28.66
*Bryodema hyalinala* Zheng&Zhang, 1981	Guanjiao Beach, Tianjun County, Qinghai	98.91	37.22
*Bryodema pseudohyalinala* Zheng, Lin&Zhang, 2012	Langma, Gongba County, Tibet	88.51	28.27
*Bryodema kangmarensis* Zheng, Lin&Zhang, 2012	Kangma Gala, Tibet	89.68	28.56
*Bryodema nigristria* Zheng&Chen, 2001	Yushu, Qinghai	97.01	32.99
*Bryodema gebleri gebleri* (Fischer von Waldheim, 1836)	Zhimen Da, Yushu, Qinghai	97.26	33.01
	Yushu, Qinghai	97.01	32.99
*Bryodema gansuensis* Zheng, 1985	Diebu County, Gansu	103.22	34.06
*Bryodema ochropenna* Zheng&Xi, 1985	Dachagai Ranch, Subei Mongol Autonomous County, Gansu	99.51	38.66
*Bryodema qilianshanicum* Lian&Zheng, 1984	Xining, Qinghai	101.78	36.62
	Datong Hui and Tu Autonomous County, Qinghai	101.69	36.93
	Ping’an County, Qinghai	102.11	36.50
	Huzhu Tuzu Zizhixian, Qinghai	101.96	36.84
	Menyuan Hui Autonomous County, Qinghai	101.61	37.39
*Angaracris barabensis* (Pallas, 1773)	Tianjun County, Qinghai	99.02	37.30
	Tongde, Qinghai	100.58	35.25
	Gonghe County, Qinghai	100.62	36.28
	Qilian County, Qinghai	100.25	38.18
	Baiyang Gully, Qilian County, Qinghai	100.28	38.24
	Gangcha County, Qinghai	100.14	37.32
	Xinghai, Qinghai	99.99	35.59
	Xunhua County, Qinghai	102.45	35.68
	Baoan, Tongren County, Qinghai	102.07	35.62
	Mai Xiu Forest Farm, Zeku County, Qinghai	101.93	35.26
	Luqu County, Gansu	102.49	34.39
	Maqu County, Gansu	102.07	34.00
	Lintan, Gansu	103.53	34.79
	Xiahe, Gansu	102.52	35.20
*Angaracris rhodopa* (Fischer von Waldheim, 1836)	Xining, Qinghai	101.78	36.62
	Daotang River, Gonghe County, Qinghai	100.97	36.40
	Gonghe Tiebujia, Qinghai	99.58	37.03
	Nian Di Township, Gonghe County, Qinghai	100.39	36.38
	Santa La, Gonghe County, Qinghai	100.27	36.06
	Ziketan, Xinghai County, Qinghai	99.98	35.59
	Dahe Dam, Xinghai, Qinghai	100.01	35.52
	Wenquan Township, Xinghai, Qinghai	99.67	35.22
	Heika Mountain, Xinghai County, Qinghai	100.05	35.78
	Batan, Qinghai	101.31	35.32
	Guinan County, Qinghai	100.74	35.57
	Guinan Mugetan, Qinghai	100.48	35.34
	Duogun Township, Tongde, Qinghai	100.37	34.94
	Qingmei Township, Tongde, Qinghai	101.21	34.82
	Guide County, Qinghai	101.43	36.04
	Xunhua County, Qinghai	102.49	35.85
	Tianjun County, Qinghai	99.02	37.30
	Huzhu Tuzu Zizhixian, Qinghai	101.96	36.84
	Datong Hui and Tu Autonomous County, Qinghai	101.69	36.93
	Ping’an County, Qinghai	102.11	36.50
	Menyuan Hui Autonomous County, Qinghai	101.61	37.39
	Lintan, Gansu	103.53	34.79
	Bai Da Ban Prairie, Qilian Mountains, Gansu	99.30	38.81
	Maqu County, Gansu	102.07	34.00
	Luqu County, Gansu	102.49	34.59
	Diebu County, Gansu	103.22	34.06
	Zhuoni County, Gansu	103.51	34.59
	Zhouqu, Gansu	104.37	33.79
	Xiahe, Gansu	102.52	35.20
*Angaracris morulimarginis* Huang, 1981	Lhasa, Tibet	91.17	29.66
	Gangcha County, Qinghai	100.14	37.32
*Angaracris acrohylinna* Bi, 1986	Lhasa, Tibet	91.17	29.66
*Angaracris nigripennis* Lian&Zheng, 1984	Xiahe, Gansu	102.52	35.20
*Uvaroviola multispinosa* Bey-Bienko, 1930	Xiahe, Gansu	102.52	35.20
	Gonghe County, Qinghai	100.22	36.13
	Tongde, Qinghai	100.58	35.25
	Maqin, Qinghai	100.24	34.48
	Tongren County, Qinghai	102.02	35.52
*Ceracris nigricornis nigricornis* Walker, 1870	Motuo, Tibet	95.33	29.33
	Zhouqu, Gansu	104.37	33.79
	Xiahe, Gansu	102.52	35.20
*Ceracris xizangensis xizangensis* Liu, 1981	Tongmai, Bomi, Tibet	95.08	30.10
	Yumei, Nyingchi, Tibet	94.95	29.59
	Zayü, Nyingchi, Tibet	95.14	29.90
*Ceracris versicolor* (Brunner von Wattenwyl, 1893)	Motuo, Tibet	95.33	29.33
*Ceracrisoides shannanensis* Bi&Xia, 1987	Shannan, Tibet	91.77	29.24
*Anaptygus qinghaiensis* Yin, 1984	Zeku, Qinghai	101.46	35.04
	Xunhua County, Qinghai	102.49	35.85
*Anaptygus yueliangwan* Mao, Ren&Ou, 2011	Deiqen Moon Bay, Yunnan	99.31	28.20
*Anaptygus yulongensis* Wang, Zheng&Lian, 2005	Jade Dragon Snow Mountain, Lijiang, Yunnan	100.17	27.10
*Anaptygus furculus* Huo&Zheng	Xunhua County, Qinghai	102.49	35.85
*Anaptygus longipennis* Wang, Zheng&Lian, 2004	Laojun Mountain, Jianchuan, Yunnan	99.59	26.56
*Anaptygus uvarovi* (Chang, 1937)	Zhongzha Town, Batang County, Sichuan	99.17	29.50
	Ganzi County, Sichuan	100.86	28.42
	Luding County, Sichuan	102.23	29.92
	Kangding, Sichuan	101.95	29.98
*Arcyptera coreana* Shiraki, 1930	Gonghe, Qinghai	100.62	36.28
	Maerkang County, Sichuan	102.20	31.91
*Leuconemacris litangensis* (Yin, 1983)	Litang County, Sichuan	100.27	30.00
*Leuconemacris xizangensis* (Yin, 1984)	Markam, Tibet	98.59	29.69
	Xiangcheng County, Sichuan	100.05	29.12
	Xiangcheng County, Sichuan	99.75	29.12
	Batang County, Sichuan	99.11	30.01
*Leuconemacris daochengensis* Zheng, 1988	Daocheng County, Sichuan	100.30	29.04
	Daocheng County, Sichuan	100.18	28.55
*Leuconemacris longipennis* Zheng, 1988	Batang County, Sichuan	99.11	30.01
*Leuconemacris breviptera* (Yin, 1983)	Litang County, Sichuan	100.27	30.00
	Yajiang, Sichuan	101.01	30.03
	Dechai Township, Yajiang, Sichuan	100.76	29.67
	Kangding, Sichuan	101.95	30.16
*Leuconemacris xiangchengensis* Zheng, 1988	Xiangcheng County, Sichuan	99.80	28.93
	Liziping Township, Muli County, Sichuan	100.29	27.97
	Muli County, Sichuan	100.86	28.42
*Leuconemacris shalulishanensis* Zheng, 1988	Daocheng County, Sichuan	100.30	29.04
*Leuconemacris hengduanshanensis* Zheng, 1988	Xiangcheng County, Sichuan	99.80	28.93
*Leuconemacris xinlongensis* Mao&Huang, 2023	Tongxiao Township, Xinlong, Sichuan	100.08	30.89
	Xinlong County, Sichuan	99.90	30.85
*Leuconemacris acuminata* Mao&Huang, 2023	Bowo Township, Muli County, Sichuan	101.12	28.78
*Leuconemacris muliensis* Mao&Huang 2023	Bowo Township, Muli County, Sichuan	101.12	28.78
	Muli County, Sichuan	101.10	29.00
*Pararcyptera microptera* Ikonnikov, 1911	Guide County, Qinghai	101.43	36.04
	Bai Da Ban Prairie, Qilian Mountains, Gansu	99.30	38.81
*Dnopherula brevicornis* (Bi&Xia, 1987)	Jilong, Tibet	85.30	28.86
*Dnopherula taeniatus* (Bolívar, 1902)	Chayu, Tibet	97.47	28.66
*Dnopherula xizangensis* (Zheng&Liping, 1986)	Chayu, Tibet	97.47	28.66
*Dnopherula yuanmowensis* (Zheng, 1977)	Aba County, Sichuan	101.70	32.90
*Dnopherula neovittatus* Otte, 1997	Chayu, Tibet	97.47	28.66
*Dnopherula sinensis* (Uvarov, 1925)	Deqin County, Yunnan	98.92	28.47
	Lanping County, Yunnan	99.42	26.46
	Ninglang County, Yunnan	100.85	27.29
*Eremippus persicus* Uvarov, 19291	Zanda, Tibet	79.80	31.48
*Eremippus simplex* (Eversmann, 1859)	Xining, Qinghai	101.78	36.62
*Eremippus heimahoensis* Zheng&Hang, 1974	Hei Ma He Township, Gonghe County, Qinghai	99.78	36.73
*Podismomorpha gibba* Lian&Zheng, 1984	Ma’ai, Luqu, Gansu	102.52	35.20
*Squamopenna gansuensis* Lian&Zheng, 1984	Ganjia Town, Xiahe County, Gansu	102.52	35.40
*Euchorthippus unicolor* (Ikonnikov, 1913)	Jianzha, Qinghai	102.03	35.94
	Guinan County, Qinghai	100.75	35.59
	Xining, Qinghai	101.78	36.62
*Euchorthippus weichowensis* Chang, 1937	Tongren County, Qinghai	103.59	31.48
*Euchorthippus flexucarinatus* Bi&Xia, 1987	Qusongbu, Tibet	80.73	31.13
	Lhünzhub County, Tibet	92.46	28.41
	Milin, Tibet	94.21	29.22
	Qusang Nama, Tibet	82.24	32.06
*Euchorthippus cheui* Hsia, 1965	Diebu County, Gansu	103.22	34.06
	Luqu, Gansu	102.49	34.59
	Zhuoni, Gansu	103.51	34.59
	Lintan, Gansu	103.35	34.69
	Xiahe, Gansu	102.52	35.20
*Euchorthippus yungningensis* Cheng&Chiu, 1965	Zhuoni, Gansu	103.51	34.59
	Diebu County, Gansu	103.22	34.06
*Eclipophleps xizangensis* Liu, 1980	Ngamring County, Tibet	88.57	29.17
*Kangacris rufipes* Yin, 1983	Litang County, Sichuan	100.27	30.00
	Kuiyong Gully, Danba County, Sichuan	101.77	30.56
	Garzê Town, Sichuan	99.99	31.63
	Kangding, Sichuan	101.95	30.16
	Pengta Township, Kangding, Sichuan	102.29	30.43
	Dechai, Yajiang, Sichuan	101.35	29.95
*Macrokangacris luteoarmilla* Yin, 1983	Batang County, Sichuan	99.11	30.01
*Kangacrisoides jiangdaensis* Zheng, Dong, Bai&Xu, 2013	Jiangda County, Tibet	98.22	31.50
*Chorthippus intermedius* (Bey-Bienko, 1926)	Kagong Township, Jiangda County, Tibet	98.14	31.44
	Guxiang, Bomi, Tibet	95.50	29.91
	Chayu, Tibet	97.47	28.66
	Basu, Tibet	96.92	30.05
	Gujing, Tibet	97.22	29.15
	Bomi, Tibet	95.77	29.86
	Riwoqe, Tibet	96.60	31.21
	Tongde, Qinghai	100.58	35.25
	Zeku, Qinghai	101.46	35.04
	Tongren County, Qinghai	102.02	35.52
	Qilian County, Qinghai	100.25	38.18
	Menyuan Hui Autonomous County, Qinghai	101.61	37.39
	Datong Hui and Tu Autonomous County, Qinghai	101.69	36.93
	Guinan County, Qinghai	100.75	35.59
	Xunhua County, Qinghai	102.49	35.85
	Huzhu Tuzu Zizhixian, Qinghai	101.96	36.84
	Nagqu, Qinghai	96.48	32.19
	Maqu County, Gansu	102.07	34.00
	Xiahe, Gansu	102.52	35.20
	Jiuzhaigou Valley, Sichuan	104.24	33.26
	Luhuo, Sichuan	100.64	31.43
	Kangding, Sichuan	101.95	30.16
	Batang County, Sichuan	99.11	30.01
*Chorthippus qingzangensis* Yin, 1984	Cháiwéi Township, Chamdo, Tibet Cháiwéi Township, Chamdo, TibeSangduo Town, Riwoqe, Tibet: Sangduo Town, Riwoqe, Tibet	97.14	31.57
	Kagong Township, Jiangda County, Tibet	98.14	31.44
	Cháiwéi Township, Chamdo, TibeSangduo Town, Riwoqe, Tibet	96.60	31.21
	Ranwu Town, Basu, Tibet	96.75	29.50
	Riwoqe, Tibet	96.60	31.21
	Chamdo, Tibet	97.17	31.15
	Basu, Tibet	96.92	30.05
	Qilian County, Qinghai	100.25	38.18
	Ping’an County, Qinghai	102.11	36.50
	Yushu, Qinghai	97.01	32.99
	Xining, Qinghai	101.78	36.62
	Datong Hui and Tu Autonomous County, Qinghai	101.69	36.93
	Ganzi County, Sichuan	99.99	31.62
	Jiuzhaigou Valley, Sichuan	104.24	33.26
*Chorthippus latisulcus* (Zheng&He, 1995)	Datong Hui and Tu Autonomous County, Qinghai	101.69	36.93
	Haiyan, Qinghai	101.00	36.90
*Chorthippus dabanshanensis* Chen, Zeng&Bao, 2011	Tianjun County, Qinghai	99.02	37.30
	Gonghe, Qinghai	100.62	36.28
	Menyuan Hui Autonomous County, Qinghai	101.61	37.39
	Huzhu Tuzu Zizhixian, Qinghai	101.96	36.84
	Daban Mountain, Qinghai	102.09	37.06
	Xunhua County, Qinghai	102.49	35.85
	Qilian County, Qinghai	100.25	38.18
*Chorthippus qilianshanensis* Zheng&Xie, 2000	Menyuan Hui Autonomous County, Qinghai	101.61	37.39
	Tianjun County, Qinghai	99.02	37.30
	Gonghe, Qinghai	100.62	36.28
	Qilian County, Qinghai	100.25	38.18
	Huzhu Tuzu Zizhixian, Qinghai	101.96	36.84
	Xunhua County, Qinghai	102.49	35.85
*Chorthippus qinghaiensis* Wang&Zheng, 1992	Qumarlêb, Qinghai	95.80	34.13
	Mǎnigān Gēng, Dégé, Sichuan	99.20	31.92
*Chorthippus jishishanensis* Zheng&Xie, 2000	Xunhua County, Qinghai	102.49	35.85
*Chorthippus guideensis* Zheng&al, 2009	Gārdé Qiānhù Village, Qinghai	101.55	36.29
*Chorthippus changtunensis* Yin, 1984	Beibeng, Motuo, Tibet	95.17	28.91
	Chamdo, Tibet	97.17	31.15
	Jiāngdá, Chamdo, Tibet	98.22	31.50
	Ganzi County, Sichuan	99.99	31.62
	Batang County, Sichuan	99.11	30.01
*Chorthippus flexivenus* (Liu, 1981)	Ranwu Lake, Basu, Tibet	96.79	29.45
	Jilong County, Shigatse, Tibet	85.30	28.86
*Chorthippus chayuensis* Yin, 1984	Linzhi Town, Tibet	94.48	29.57
	Chayu, Tibet	97.47	28.66
	Linzhi, Tibet	94.36	29.65
	Muli County, Sichuan	100.86	28.42
*Chorthippus albonemus* Zheng&Tu, 1964	Nagqu, Qinghai	96.48	32.19
	Guinan County, Qinghai	100.75	35.59
	Guide County, Qinghai	101.43	36.04
	Xining, Qinghai	101.78	36.62
	Jianzha, Qinghai	102.03	35.94
	Xunhua County, Qinghai	102.49	35.85
	Huzhu Tuzu Zizhixian, Qinghai	101.96	36.84
	Ledu District, Qinghai	102.40	36.48
	Datong Hui and Tu Autonomous County, Qinghai	101.69	36.93
	Tongren County, Qinghai	102.02	35.52
	Ping’an County, Qinghai	102.11	36.50
	Ganzi County, Sichuan	99.99	31.62
	Muli County, Sichuan	100.86	28.42
	Bai Da Ban Prairie, Qilian Mountains, Gansu	99.30	38.81
	Maqu County, Gansu	102.07	34.00
	Luqu County, Gansu	102.49	34.59
	Diebu County, Gansu	103.22	34.06
	Lintan County, Gansu	103.35	34.69
	Zhuoni County, Gansu	103.51	34.59
	Zhouqu, Gansu	104.37	33.79
	Xiahe, Gansu	102.52	35.20
*Chorthippus chinensis* (Tarbinsky, 1927)	Xiahe, Gansu	102.52	35.20
	Ganzi County, Sichuan	99.99	31.62
	Muli County, Sichuan	100.86	28.42
	Jiuzhaigou Valley, Sichuan	103.95	33.30
*Chorthippus hsiai* Cheng&Tu, 1964	Litang County, Sichuan	100.27	30.00
	Xining, Qinghai	101.78	36.62
	Guinan County, Qinghai	100.75	35.59
	Gonghe County, Qinghai	100.62	36.28
	Xinghai, Qinghai	99.99	35.59
	Jianzha, Qinghai	102.03	35.94
	Tongde, Qinghai	100.58	35.25
	Guide County, Qinghai	101.43	36.04
	Tongren County, Qinghai	102.02	35.52
	Maqu County, Gansu	102.07	34.00
	Luqu County, Gansu	102.49	34.59
	Diebu County, Gansu	103.22	34.06
	Lintan County, Gansu	103.35	34.69
	Zhuoni County, Gansu	103.51	34.59
	Zhouqu, Gansu	104.37	33.79
	Xiahe, Gansu	102.52	35.20
*Chorthippus longisonus* (Li&Yin, 1987)	Xunhua County, Qinghai	102.49	35.85
	Litang, Sichuan	100.12	29.83
*Chorthippus markamensis* (Yin, 1984)	Markam, Tibet	98.59	29.69
	Jiangda County, Tibet	98.22	31.50
*Chorthippus biguttulus biguttulus* (Linnaeus, 1758)	Chamdo, Tibet	97.17	31.15
	Riwoqe, Tibet	96.60	31.21
	Chayab, Tibet	97.57	30.66
	Kagong Township, Jiangda County, Tibet	98.14	31.44
	Zhiduo, Qinghai	95.62	33.84
	Yushu, Qinghai	97.01	32.99
	Gonghe County, Qinghai	100.62	36.28
	Xinghai, Qinghai	99.99	35.59
	Tianjun County, Qinghai	99.02	37.30
	Luqu, Gansu	102.49	34.39
	Zhuoni, Gansu	103.51	34.59
*Chorthippus chapini* Chang, 1939	Kangding, Sichuan	101.95	30.16
	Luding County, Sichuan	102.23	29.92
	Garzê Town, Sichuan	99.99	31.63
*Chorthippus grahami* Chang, 1937	Maerkang County, Sichuan	102.20	31.91
	Xiaojin, Sichuan	102.36	31.00
	Jinchuan County, Sichuan	102.06	31.48
	Batang County, Sichuan	99.11	30.01
*Chorthippus brevipterus* Yin, 1984	Aba County, Sichuan	101.70	32.90
	Mai Xiu Forest Farm, Zeku County, Qinghai	101.93	35.26
	Jiuzhi, Qinghai	101.48	33.43
*Chorthippus fallax* (Zubovsky), 1899	Xiahe, Gansu	102.52	35.20
	Maqu County, Gansu	102.07	34.00
	Luqu County, Gansu	102.49	34.59
	Diebu County, Gansu	103.22	34.06
	Lintan County, Gansu	103.35	34.69
	Zhuoni County, Gansu	103.51	34.59
	Zhouqu, Gansu	104.37	33.79
	Bai Da Ban Prairie, Qilian Mountains, Gansu	99.30	38.81
	Ganzi County, Sichuan	99.99	31.62
	Muli County, Sichuan	100.86	28.42
	Daotang River, Gonghe County, Qinghai	100.97	36.40
	Hei Ma He Township, Gonghe County, Qinghai	99.78	36.73
	Gonghe Tiebujia, Qinghai	99.58	37.03
	Nian Di Township, Gonghe County, Qinghai	100.39	36.38
	Santa La, Gonghe County, Qinghai	100.27	36.06
	Heika Mountain, Xinghai County, Qinghai	100.05	35.78
	Guinan County, Qinghai	100.74	35.57
	Mùgé Tān, Guinan County, Qinghai	100.83	35.64
	Guinan Mugetan, Qinghai	100.48	35.34
	Mai Xiu Forest Farm, Zeku County, Qinghai	101.93	35.26
	Xining, Qinghai	101.78	36.62
	Huangyuan County, Qinghai	101.26	36.68
	Jianzha, Qinghai	102.03	35.94
	Ping’an County, Qinghai	102.11	36.50
	Ledu District, Qinghai	102.40	36.48
	Guide County, Qinghai	101.43	36.04
	Maqin, Qinghai	100.24	34.48
	Menyuan Hui Autonomous County, Qinghai	101.61	37.39
	Xunhua County, Qinghai	102.49	35.85
	Datong Hui and Tu Autonomous County, Qinghai	101.69	36.93
	Tongren County, Qinghai	102.02	35.52
	Huzhu Tuzu Zizhixian, Qinghai	101.96	36.84
*Chorthippus brunneus huabeiensis* Xia&Jin, 1982	Ledu District, Qinghai	102.40	36.48
	Jianzha, Qinghai	102.03	35.94
	Xiahe, Gansu	102.52	35.20
	Bai Da Ban Prairie, Qilian Mountains, Gansu	99.30	38.81
	Maqu County, Gansu	102.07	34.00
	Diebu County, Gansu	103.22	34.06
	Lintan County, Gansu	103.35	34.69
	Zhuoni County, Gansu	103.51	34.59
	Zhouqu, Gansu	104.37	33.79
	Luqu, Gansu	102.49	34.59
	Kangding, Sichuan	101.95	30.16
	Maerkang County, Sichuan	102.20	31.91
*Chorthippus maerkangensis* (Zheng, 1980)	Maerkang County, Sichuan	102.20	31.91
	Jinchuan County, Sichuan	102.06	31.48
*Chorthippus xiangchengensis* (Liu, 1985)	Xiangcheng County, Sichuan	99.80	28.93
*Chorthippus louguanensis* Zheng&Tu, 1964	Jinchuan County, Sichuan	102.06	31.48
	Xiahe, Gansu	102.52	35.20
	Diebu County, Gansu	103.22	34.06
	Luqu County, Gansu	102.49	34.59
	Zhuoni County, Gansu	103.51	34.59
	Maqu County, Gansu	102.48	34.59
	Lintan County, Gansu	103.35	34.69
*Chorthippus occidentalis* Xia&Jin, 1982	Maerkang County, Sichuan	102.20	31.91
	Xiaojin, Sichuan	102.36	31.00
	Kangding, Sichuan	101.95	30.16
	Jinchuan County, Sichuan	102.06	31.48
*Chorthippus squamopennis* Zheng, 1980	Jinchuan County, Sichuan	102.06	31.48
*Chorthippus dubius* (Zubovski, 1898)	Daotang River, Gonghe County, Qinghai	100.97	36.40
	Hei Ma He Township, Gonghe County, Qinghai	99.78	36.73
	Ziketan, Xinghai County, Qinghai	99.98	35.59
	Batan, Qinghai	101.31	35.32
	Guinan Mugetan, Qinghai	100.48	35.34
	Tongde, Qinghai	100.58	35.25
	Tianjun County, Qinghai	99.02	37.30
	Guide County, Qinghai	101.43	36.04
	Bai Da Ban Prairie, Qilian Mountains, Gansu	99.30	38.81
	Maqu County, Gansu	102.07	34.00
	Luqu County, Gansu	102.49	34.59
	Diebu County, Gansu	103.22	34.06
	Lintan County, Gansu	103.35	34.69
	Zhuoni County, Gansu	103.51	34.59
	Zhouqu, Gansu	104.37	33.79
	Xiahe, Gansu	102.52	35.20
*Chorthippus xiningensis* Zheng&Chen, 2001	Xishan, Xining, Qinghai	101.57	36.49
*Chorthippus haibeiensis* Zheng&Chen, 2001	Dauyinshan, Qilian County, Qinghai	100.21	38.15
*Chorthippus helanshanensis* Zheng, 1999	Nagqu, Qinghai	96.49	32.21
*Chorthippus rufitibis* (Zheng, 1989)	Ping’an County, Qinghai	102.11	36.50
	Huzhu Tuzu Zizhixian, Qinghai	101.96	36.84
	Xunhua County, Qinghai	102.49	35.85
*Chorthippus brunneus brunneus* (Thunbeng, 1815)	Jianzha, Qinghai	102.03	35.94
	Jiangda County, Tibet	98.22	31.50
	Riwoqe, Tibet	96.60	31.21
	Chamdo, Tibet	97.17	31.15
	Bomi, Tibet	95.77	29.86
	Xiahe, Gansu	102.52	35.20
*Chorthippus xunhuaensis* Zheng&Xie, 2000	Xunhua County, Qinghai	102.49	35.85
*Chorthippus leduensis* Zheng&Xin, 1999	Ledu District, Qinghai	102.40	36.48
*Chorthippus gongbuensis* Liang&Zheng, 1991	Gongbujiangda, Tibet	93.24	29.89
*Chorthippus mollis* (Charpentieri, 1825)	Jiangda County, Tibet	98.22	31.50
	Ganzi County, Sichuan	99.99	31.62
*Chorthippus amplintersitus* Liu, 1981	Riwoqe, Tibet	96.60	31.21
	Xiangcheng County, Sichuan	99.72	29.11
*Chorthippus rubensabdomenis* Liu, 1981	Jilong, Tibet	85.30	28.86
	Mai Xiu Forest Farm, Zeku County, Qinghai	101.93	35.26
	Daocheng County, Sichuan	100.29	28.72
*Chorthippus hemipeterus* Uvarov, 1926	Qusong, Zanda, Tibet	79.18	32.20
*Chorthippus flavabdomenis* Liu, 1981	Jitang, Chayab, Tibet	97.35	30.74
*Chorthippus tibetanus* Uvarov, 1935	Shannan City, Tibet	91.77	29.24
	Linzhi, Tibet	94.36	29.65
	Chamdo, Tibet	97.17	31.15
	Bomi, Tibet	95.77	29.86
*Chorthippus separatanus* Liu, 1981	Jigong, Chayu, Tibet	97.07	28.84
*Chorthippus aethalinus* (Zubovski, 1899)	Xiahe, Gansu	102.52	35.20
*Chorthippus laifoveatus* Xia&Jin, 1982	Motuo, Tibet	95.33	29.33
*Chorthippus dichrous* (Eversmann, 1859)	Huzhu Tuzu Zizhixian, Qinghai	101.96	36.84
*Chorthippus huchengensis* Xia&Jin, 1982	Datong, Xining, Qinghai	101.69	36.93
	Qilian County, Qinghai	100.25	38.18
	Geermu, Qinghai	94.90	36.40
	Guide County, Qinghai	101.43	36.04
	Jianzha, Qinghai	102.03	35.94
	Tongren County, Qinghai	102.02	35.52
	Lazikou, Diebu County, Gansu	103.90	34.06
*Chorthippus daitongensis* Huo, 1994	Haiyan, Qinghai	101.00	36.90
*Chorthippus fusigeniculatus* Zheng&Men, 2008	Chamdo, Tibet	97.17	31.15
*Chorthippus xueshanensis* Zheng&Mao, 1997	Lanping County, Yunnan	99.42	26.46
	Shangri-La, Yunnan	99.74	27.84
	Laojun Mountain, Jianchuan, Yunnan	99.59	26.56
	Ninglang County, Yunnan	100.85	27.29
	Hengduan Mountains, Deqin County, Yunnan	98.99	27.58
	Yanyuan County, Sichuan	101.51	27.43
*Chorthippus deqinensis* Liu, 1984	Deqin County, Yunnan	98.92	28.47
*Chorthippus nemus* Liu, 1984	Shangri-La, Yunnan	99.74	27.84
	Chongjiang River, Shangri-La, Yunnan	100.01	27.27
	Deqin County, Yunnan	98.92	28.47
*Chorthippus yajiangensis* Zheng&Shi, 2007	Dechai, Yajiang, Sichuan	101.35	29.95
*Chorthippus kangdingensis* Zheng&Shi, 2007	Pengta, Kangding, Sichuan	102.29	30.43
*Chorthippus neipopennis* Xia&Jin, 1982	Xi’e Luo, Sichuan	100.72	30.02
*Chorthippus anomopterus* Liu, 1984	Deqin County, Yunnan	98.92	28.47
*Chorthippus yanyuanensis* Jin&Lin, 1982–1983	Yanyuan County, Sichuan	101.39	27.69
	Hongxing Township, Ruoergai County, Sichuan	102.74	34.10
*Omocestus cuonaensis* Yin, 1984	Qingduo Township, Bomi, Tibet	95.60	30.06
	Guxiang, Bomi, Tibet	95.50	29.91
	Ranwu Town, Basu, Tibet	96.75	29.50
	Milin, Tibet	94.21	29.22
	Nyingchi, Tibet	94.36	29.65
	Lang County, Tibet	93.07	29.05
	Cona, Tibet	91.96	28.00
*Omocestus gonggarensis* Zheng&Chen, 1995	Gonggar County, Tibet	90.98	29.29
*Omocestus haemorrhoidalis* (Charpentier, 1825)	Kagong Township, Jiangda County, Tibet	98.14	31.44
	Chamdo, Tibet	97.17	31.15
	Riwoqe, Tibet	96.60	31.21
	Datong County, Xining, Qinghai	101.69	36.93
	Yushu, Qinghai	97.01	32.99
	Tongde, Qinghai	100.58	35.25
	Tongren County, Qinghai	102.02	35.52
	Guinan County, Qinghai	100.75	35.59
	Zeku, Qinghai	101.46	35.04
	Qilian County, Qinghai	100.25	38.18
	Litang County, Sichuan	100.27	30.00
	Ri’erlang Mountain, Ruoergai County, Sichuan	102.79	34.03
	Mǎnigān Gēng, Dégé, Sichuan	99.20	31.92
	Ganzi County, Sichuan	99.99	31.62
	Xiahe, Gansu	102.52	35.20
	Lintan, Gansu	103.53	34.79
	Diebu County, Gansu	103.22	34.06
	Zhuoni County, Gansu	103.51	34.59
	Maqu County, Gansu	102.07	34.00
	Luqu, Gansu	102.49	34.59
*Omocestus avellaeusitibia* Zheng, Dong&Xu, 2013	Ganzi County, Sichuan	99.99	31.62
*Omocestus petraeus* (Brisout de Barneville, 1856)	Dechai, Yajiang, Sichuan	101.35	29.95
	Menyuan Hui Autonomous County, Qinghai	101.61	37.39
	Menyuan Hui Autonomous County, Qinghai	101.62	37.37
*Omocestus nyalamus* Xia, 1981	Damxung County, Tibet	91.10	30.49
	Nyalam, Tibet	85.98	28.16
	Dingri County, Tibet	87.12	28.66
	Lazi County, Tibet	87.64	29.08
*Omocestus megaoculus* Yin, 1984	Nyalam, Tibet	86.04	28.52
	Markam, Tibet	98.59	29.69
*Omocestus motuoensis* Yin, 1984	Motuo, Tibet	95.33	29.33
	Milin, Tibet	94.21	29.22
	Bomi, Tibet	95.77	29.86
	Delomagnigou, Sichuan	99.20	31.92
*Omocestus pinanensis* Zheng&Xie, 2001	Ping’an County, Qinghai	102.11	36.50
*Omocestus qinghaihuensis* Zheng&Xie, 2001	South Shore of Qinghai Lake, Qinghai	100.54	36.56
	Tianjun County, Qinghai	99.02	37.30
	Gonghe, Qinghai	100.62	36.28
	Guide County, Qinghai	101.43	36.04
	Guinan County, Qinghai	100.75	35.59
	Tongde, Qinghai	100.58	35.25
	Huzhu Tuzu Zizhixian, Qinghai	101.96	36.84
*Omocestus hingstoni* Uvarov, 1925	Dongjiu, Nyingchi, Tibet	94.80	29.94
	Mount Qomolangma, Tibet	86.86	28.13
*Omocestus enitor* Uvarov, 1924	Lhünzhub County, Tibet	92.46	28.41
*Omocestus laojunshanensis* Mao&Xu, 2004	Laojun Mountain, Jianchuan, Yunnan	99.59	26.56
	Jade Dragon Snow Mountain, Lijiang, Yunnan	100.18	27.10
*Nivisacris zhongdianensis* Liu, 1984	Shangri-La, Yunnan	99.74	27.84
*Saxetophilus qinghaiensis* Zheng, Chen&Li, 2012	Kunlun Mountains, Xinjiang	81.67	35.47
	Qumarlêb, Qinghai	95.80	34.13
	Hei Ma He Township, Gonghe County, Qinghai	99.78	36.73
	Nian Di Township, Gonghe County, Qinghai	100.39	36.38
	Wenquan Township, Xinghai, Qinghai	99.67	35.22
	Heika Mountain, Xinghai County, Qinghai	100.05	35.78
	Tongde, Qinghai	100.58	35.25
	Maqin, Qinghai	100.24	34.48
	Qilian County, Qinghai	100.25	38.18
	Xining, Qinghai	101.78	36.62
	Jianzha, Qinghai	102.03	35.94
	Guinan County, Qinghai	100.75	35.59
	Guide County, Qinghai	101.43	36.04
*Saxetophilus petulans* Umnov, 1930	Luqu, Gansu	102.49	34.59
	Xiahe, Gansu	102.52	35.20
*Saxetophilus gansuensis* Wang, Zheng&Lian, 2006	Luqu, Gansu	102.49	34.59
*Oreoptygonotus chinghaiensis* (Zheng&Hang, 1974)	Madoi County, Golog Tibetan Autonomous Prefecture, Qinghai	98.21	34.92
	Guinan County, Qinghai	100.75	35.59
	Tongde, Qinghai	100.58	35.25
	Hei Ma He Township, Gonghe County, Qinghai	99.78	36.73
	Heka Mountain, Xinghai, Qinghai	100.05	35.78
	Tongren County, Qinghai	102.02	35.52
	Zeku, Qinghai	101.46	35.04
	Henan, Qinghai	101.62	34.73
	Maqin, Qinghai	100.24	34.48
	Tianjun County, Qinghai	99.02	37.30
*Oreoptygonotus tibetanus* Tarbinsky, 1927	Guinan County, Qinghai	100.75	35.59
	Tongde, Qinghai	100.58	35.25
	Eling Lake, Qinghai	97.74	34.92
	Zaling Lake, Qinghai	97.91	35.08
	Madoi County, Qinghai	98.21	34.91
	Qumarlêb, Qinghai	95.80	34.13
*Oreoptygonotus brachypterus* Yin, 1984	Heka Mountain, Xinghai, Qinghai	100.05	35.78
*Oreoptygonotus robustus* Yin, 1984	Zeku, Qinghai	101.46	35.04
	Henan, Qinghai	101.62	34.73
	Jiangda County, Tibet	98.10	31.80
*Oreoptygonotus nigrofasciatus* Yin, 1979	Gaize County, Tibet	84.68	32.24
*Oreoptygonotus belonocercus* Liu, 1981	Cuomei County, Tibet	91.43	28.42
*Ruganotus rufipes* Yin, 1979	Pulan County, Tibet	81.18	30.29
*Ptygonotus sichuanensis* Zheng, 1983	Jiuzhaigou Valley, Sichuan	104.24	33.26
	Lixian County, Sichuan	103.16	31.44
	Qingzhenzhan Mountain, Gade County, Qinghai	100.12	34.17
	Batang, Yushu, Qinghai	97.15	32.83
*Ptygonotus semenovi semenovi* Tarbinsky, 1927	Songpan County, Sichuan	103.60	32.64
*Ptygonotus tarbinskyi* Uvarov, 1930	Songpan County, Sichuan	103.60	32.64
	Ma’ai, Luqu, Gansu	102.52	35.20
	Xiahe, Gansu	102.52	35.20
	Diebu County, Gansu	103.21	34.05
*Ptygonotus gurneyi* Chang, 1937	Kangding, Sichuan	101.95	30.16
	Dechai, Yajiang, Sichuan	101.35	29.95
*Ptygonotus chinghaiensis* Ying, 1974	Tongde, Qinghai	100.58	35.25
	Guinan County, Qinghai	100.75	35.59
*Ptygonotus hocashanensis* Cheng&Hang, 1974	Heika Mountain, Xinghai County, Qinghai	100.05	35.78
	Luqu, Gansu	102.49	34.59
	Xiahe, Gansu	102.52	35.20
	Maqu County, Gansu	102.07	34.00
	Diebu County, Gansu	103.22	34.06
*Ptygonotus brachypterus* Yin, 1974	Yushu, Qinghai	97.01	32.99
	Zhiduo, Qinghai	95.62	33.84
	Amdo, Tibet	91.68	32.26
*Ptygonotus gansuensis* Zheng&Chang, 1994	Diebu County, Gansu	103.22	34.06
*Ptygonotus semenovi antennatus* Mistshenko, 1951	Luqu, Gansu	102.49	34.59
*Ptygonotus rangkouensis* Zheng&Xu, 2009	Raokou Township, Sichuan	102.50	32.21
*Ptygonotus anurous* Zheng&Xu, 2009	Heishui County, Sichuan	102.99	32.06
*Asulconotoides sichuanensis* Liu, 1984	Daocheng County, Sichuan	100.10	29.00
	Haizi Mountain, Litang, Sichuan	100.10	29.38
	Hengduan Mountains, Yunnan	99.00	27.50
*Asulconotoides asulcata* (Zheng, 1988)	Batang County, Sichuan	99.11	30.01
*Asulconotoides microptera* (Zheng, 1988)	Batang County, Sichuan	99.11	30.01
*Asulconotus chinghaiensis* Ying, 1974	Duocai, Zhiduo, Qinghai	95.42	33.81
	Gande County, Guoluo, Qinghai	99.90	33.97
	Yushu, Qinghai	97.01	32.99
	Jiuzhi, Qinghai	101.48	33.43
	Qumarlêb, Qinghai	95.80	34.13
	Maqin, Qinghai	100.24	34.48
	Zadoi, Qinghai	95.30	32.89
	Aba County, Sichuan	101.70	32.90
*Asulconotus jiuzhiensis* Yin, Zheng&Yin, 2012	Jiuzhi, Qinghai	101.48	33.43
*Asulconotus kozlovi* Mistshenko, 1981	Yequ River, Yushu, Qinghai	95.57	34.22
	Madoi County, Qinghai	98.21	34.91
	Qumarlêb, Qinghai	95.80	34.13
*Asonus brachypterus* (Ying, 1974)	Zhiduo, Qinghai	95.61	33.87
	Racecourse, Yushu, Qinghai	96.96	33.00
	Duocai, Zhiduo, Qinghai	95.42	33.81
	Zhiduo, Qinghai	95.62	33.84
	Yushu, Qinghai	97.01	32.99
	Amdo, Tibet	91.68	32.26
	Nagqu City, Tibet	92.05	31.48
	Riwoqe, Tibet	96.60	31.21
*Asonus amplifurculus* Chen, Zheng&Zeng, 2010	Gārdé Qiānhù Village, Qinghai	101.55	36.29
*Asonus microfurculus* Yin, 1984	Kagila Mountain, Chamdo, Tibet	96.40	31.60
*Asonus parvoculus* Xie & Liu, 1998	Muli County, Sichuan	101.28	27.93
*Asonus qinghaiensis* Liu, 1986	Yushu, Qinghai	97.01	32.99
*Asonus longisulcus* Yin, 1984	Riwoqe, Tibet	96.60	31.21
*Pseudoasonus yushuensis* Yin, 1982	Qumarlêb, Qinghai	95.80	34.13
	Zadoi, Qinghai	95.30	32.89
	Nagqu, Qinghai	96.49	32.21
	Gade, Qinghai	99.90	33.97
	Maqin, Qinghai	100.24	34.48
	Tongde, Qinghai	100.58	35.25
	Jiuzhi, Qinghai	101.48	33.43
	Yushu, Qinghai	97.01	32.99
	Duocai, Zhiduo, Qinghai	95.42	33.81
*Pseudoasonus kangdingensis* Yin, 1983	Kangding, Sichuan	101.95	30.16
	Xinduqiao, Sichuan	101.49	30.05
*Pseudoasonus baiyuensis* Zheng, 1990	Baiyu County, Sichuan	98.82	31.21
	Hengduan Mountains, Yunnan	99.00	27.50
*Pseudoasonus orthomarginis* Zheng, Chen&Lin, 2012	Mozhugongka County, Tibet	95.42	33.81
*Transtympanacris xueshanensis* Lian&Zheng, 1985	Kangding, Sichuan	101.95	30.16
*Transtympanacris yajiangensis* Zheng, 1995	Yajiang, Sichuan	101.01	30.03
*Hypernephia everesti* Uvarov, 1922	Mount Qomolangma, Tibet	86.86	28.13
*Hypernephia xizangensis* Zheng, 1980	Gar County, Tibet	80.09	32.49
*Atympanum nigrofasciatum* Yin, 1984	Yangbajing, Tibet	90.54	30.10
	Amdo, Tibet	91.68	32.26
	Gaize County, Tibet	84.05	32.30
	Damxung County, Tibet	91.09	30.47
*Atympanum gonggarensis* Zheng&Chen, 1995	Gonggar County, Tibet	90.98	29.29
*Atympanum belonocercum* (Liu, 1981)	Karila Mountain Pass, Cuomei, Tibet	91.48	28.51
	Yangbajing, Tibet	90.54	30.10
*Atympanum antennatum* Yin, 1984	Nedong County, Tibet	91.76	29.22
*Atympanum carinotus* (Yin, 1979)	Gaize County, Tibet	84.05	32.30
	Shenzha County, Tibet	88.71	30.93
*Atympanum comainensis* (Liu), 1981	Karila Mountain Pass, Cuomei, Tibet	91.48	28.51
*Oknosacris gyirogensis* Liu, 1981	Cuoluo Long, Jilong, Tibet	85.42	29.13
*Hebetacris amplinota* Liu, 1981	Cuoluo Long, Jilong, Tibet	85.42	29.13
*Dysanema brevitibia* Niu, Zheng&Lin, 2015	Cuoqin County, Tibet	85.15	31.02
*Dysanema irvinei* Uvarov, 1925	Mount Qomolangma, Tibet	86.86	28.13
	Dingjie County, Tibet	87.76	28.37
	Gamba County, Tibet	88.51	28.27
	Lhünzhub County, Lin Karzong, Tibet	90.27	28.82
	Lhünzhub County, Lin Karzong, Tibet	89.15	27.72
*Dysanema malloryi* Uvarov, 1925	Lhünzhub County, Lin Karzong, Tibet	89.15	27.72
*Dysanema magna* Zheng, Lin, Zhang&Zeng, 2014	Dingri County, Tibet	87.12	28.66
*Stristernum ratogensis* Liu, 1981	Rutog County, Dogmarsum, Tibet	80.38	33.71
*Aeropus licenti* Chang, 1939	Heika Mountain, Xinghai County, Qinghai	100.05	35.78
	Guinan County, Qinghai	100.75	35.59
	Qilian County, Qinghai	100.25	38.18
	Dongliugou, Sunan, Gansu	99.64	38.77
	Xiahe, Gansu	102.52	35.20
	Diebu County, Gansu	103.22	34.06
	Ranwu Town, Basu, Tibet	96.75	29.50
	Zongbala Mountain, Markam, Tibet	98.62	29.72
	Cháiwéi Township, Chamdo, TibeSangduo Town, Riwoqe, Tibet	96.60	31.21
	Jiangtang Village, Sangri County, Tibet	91.98	29.25
	Gartok Town, Markam, Tibet	98.59	29.68
	Ganzi County, Sichuan	99.99	31.62
*Aeropus tibetanus* Uvarov, 1935	Ranwu Town, Tibet	96.75	29.51
	Jida Township, Basu, Tibet	96.68	29.91
	Kagong Township, Jiangda County, Tibet	98.14	31.44
	Gartok Town, Markam, Tibet	98.59	29.68
	Zuogong, Tibet	97.84	29.67
	Markam, Tibet	98.59	29.69
	Jiangda County, Tibet	98.22	31.50
	Riwoqe, Tibet	96.60	31.21
	Basu, Tibet	96.92	30.05
	Xinghai, Qinghai	99.99	35.59
	Gonghe, Qinghai	100.62	36.28
	Guinan County, Qinghai	100.75	35.59
*Gomphoceroides flavutibia* Zheng&Chen, 2001	Menyuan Hui Autonomous County, Qinghai	101.59	37.32
*Dasyhippus barbipes* (Fischer von Waldheim, 1846)	Daotang River, Gonghe County, Qinghai	100.97	36.40
	Guinan County, Qinghai	100.75	35.59
	Xinghai, Qinghai	99.99	35.59
*Myrmeleotettix palpalis* Zubovski, 1900	Quanji, Gangcha County, Qinghai	99.88	37.27
	Heika Mountain, Xinghai County, Qinghai	100.05	35.78
	Dahe Dam, Xinghai, Qinghai	100.01	35.52
	Hei Ma He Township, Gonghe County, Qinghai	99.78	36.73
	Gonghe Tiebujia, Qinghai	99.58	37.03
	Santa La, Gonghe County, Qinghai	100.27	36.06
	Nian Di Township, Gonghe County, Qinghai	100.39	36.38
	Batan, Qinghai	101.31	35.32
	Mùgé Tān, Guinan County, Qinghai	100.83	35.64
	Tongde, Qinghai	100.58	35.25
	Zhiduo, Qinghai	95.61	33.87
	Tongren County, Qinghai	102.02	35.52
	Qilian County, Qinghai	100.25	38.18
	Yushu, Qinghai	97.01	32.99
	Menyuan Hui Autonomous County, Qinghai	101.61	37.39
	Haiyan, Qinghai	101.00	36.90
	Geermu, Qinghai	94.90	36.40
	Zhiduo, Qinghai	95.62	33.84
	Tianjun County, Qinghai	99.02	37.30
	Xining, Qinghai	101.78	36.62
	Quanji, Gangca County, Qinghai	99.88	37.27
	Bai Da Ban Prairie, Qilian Mountains, Gansu	99.30	38.81
	Diebu County, Gansu	103.22	34.06
	Maqu County, Gansu	102.07	34.00
	Zhuoni County, Gansu	103.51	34.59
	Xiahe, Gansu	102.52	35.20
*Myrmeleotettix kunlunensis* Huang, 1987	Dongkunlun Karl Cave, Ruoqiang County, Xinjiang	89.03	38.20
*Aeropedelloides tibetanus* Uvarov, 1939	Zuogong, Tibet	97.84	29.67
	Bianba, Tibet	94.71	30.93
	Basu, Tibet	96.92	30.05
*Aeropedelloides altissimus* Liu, 1981	Yushu, Qinghai	97.01	32.99
	Riwoqe, Tibet	96.60	31.21
	Basu, Tibet	96.92	30.05
	Chayab, Tibet	97.57	30.66
*Aeropedelloides nigrocaudus* Liu, 1981	Chayab, Tibet	97.57	30.66
*Aeropedellus zadoensis* Yin, 1984	Zadoi, Qinghai	97.01	32.99
	Yushu, Qinghai	97.01	32.99
	Qumarlêb, Qinghai	95.80	34.13
	Nagqu, Qinghai	96.49	32.21
	Gade, Qinghai	99.90	33.97
	Maqin, Qinghai	100.24	34.48
	Tongde, Qinghai	100.58	35.25
	Jiuzhi, Qinghai	101.48	33.43
*Aeropedellus changtunensis* (Yin, 1984)	Chamdo, Tibet	97.17	31.15
*Aeropedellus prominemarginis* Zheng, 1981	Yushu, Qinghai	97.01	32.99
	Zhiduo, Qinghai	95.62	33.84
	Qumarlêb, Qinghai	95.80	34.13
	Zadoi, Qinghai	95.30	32.89
	Nagqu, Qinghai	96.49	32.21
	Gade, Qinghai	99.90	33.97
	Maqin, Qinghai	100.24	34.48
	Tongde, Qinghai	100.58	35.25
	Jiuzhi, Qinghai	101.48	33.43
*Orinhippus tibetanus* Uvarov, 1921	Bailang County, Tibet	89.26	29.11
	Lumbu Monastery, Jiangzi County, Tibet	89.73	28.86
	Dingri County, Tibet	87.12	28.66
	Sar Township, Shigatse, Tibet	87.74	28.19
	Dingjie County, Shigatse, Tibet	87.76	28.37
	Dingri, Shigatse, Tibet	88.85	29.00
	Lazi County, Tibet	87.64	29.08
	Mount Qomolangma, Tibet	86.86	28.13
*Orinhippus trisulcus* Yin, 1984	Lazi County, Tibet	87.64	29.08
	Yamdrok Lake, Tibet	90.71	28.92
	Dongga Township, Shigatse, Tibet	88.83	29.36
*Pacris xizangensis* Zhang, Zhang&Yin, 2012	Damxung County, Tibet	91.09	30.47
*Foveolatacris qinghaiensis* (Yin, 1984)	Tongde, Qinghai	100.58	35.25
*Mongolotettix japonicus* (Bolívar, 1898)	Qingzhenzhan Mountain, Gade County, Qinghai	100.12	34.17
	Huzhu Tuzu Zizhixian, Qinghai	101.96	36.84
*Mongolotettix anomopterus* (Caudell, 1921)	Diebu County, Gansu	103.22	34.06
	Zhouqu, Gansu	104.37	33.79
*Dianacris choui* Yin, 1983	Jade Dragon Snow Mountain, Lijiang, Yunnan	100.17	27.10
*Pseudoeoscyllina golmudensis* Zheng&Chen, 2012	Geermu, Qinghai	94.90	36.40
	Dulan County, Qinghai	98.10	36.30
*Carinulaenotus motuoensis* Yin, 1982	Motuo, Tibet	95.33	29.33
*Phlaeoba sikkimensis* Ramme, 1941	Motuo, Tibet	95.33	29.33
	Xiachayu, Chayu County, Tibet	97.02	28.50
	Chayu, Tibet	97.47	28.66
*Phlaeoba medogensis* Liu, 1981	Motuo, Tibet	95.33	29.33
*Phlaeoba tenebrosa* (Walker, 1871)	Motuo, Tibet	95.33	29.33
	Chayu, Tibet	97.40	28.83
*Phlaeoba albonema* Zheng, 1981	Motuo, Tibet	95.33	29.33
*Phlaeoba infumata* Brunner von Wattenwyl, 1893	Weixi County, Yunnan	99.29	27.18
*Chlorophlaeoba nigripennis* Zheng, Lin, Xu&Ma, 2013	Motuo, Tibet	95.33	29.33
*Sikkimiana darjeelingensis* (Bolívar, 1914)	Xiachaiyu, Tibet	97.02	28.50
	Motuo, Tibet	95.33	29.33
	Jilong, Tibet	85.30	28.86
*Acrida kozlovi* Mishchenko, 1951	Jianzha, Qinghai	102.03	35.94
	Xunhua County, Qinghai	102.49	35.85
	Guide County, Qinghai	101.43	36.04
	Hualong Hui Autonomous County, Qinghai	102.26	36.09
*Acrida curticnema* Huang, 1981	Motuo, Tibet	95.33	29.33
	Xiachayu, Chayu County, Tibet	97.02	28.50
	Chiba Gully, Chayu, Tibet	97.07	28.58
*Acrida cinerea* (Thunberg, 1815)	Zhouqu, Gansu	104.37	33.79
	Xiachayu, Chayu County, Tibet	97.02	28.50
*Acrida oxycephala* (Pallas, 1771)	Ledu District, Qinghai	102.40	36.48
*Acrida exaltata* (Walker, 1859)	Chayu, Tibet	97.47	28.66
*Acrida willemsei* Dirsh, 1954	Lhasa, Tibet	91.12	29.65
*Gonista chayuensis* Yin, 1984	Chayu, Tibet	97.47	28.66
	Xiachayu, Chayu County, Tibet	97.02	28.50
*Gonista bicolor* (De Hann, 1842)	Motuo, Tibet	95.33	29.33
